# Rehabilitating drug-induced long-QT promoters: *In-silico* design of hERG-neutral cisapride analogues with retained pharmacological activity

**DOI:** 10.1186/2050-6511-15-14

**Published:** 2014-03-08

**Authors:** Serdar Durdagi, Trevor Randall, Henry J Duff, Adam Chamberlin, Sergei Y Noskov

**Affiliations:** 1 Centre for Molecular Simulations and Department of Biological Sciences, University of Calgary, Calgary, Alberta, Canada; 2 Department of Biophysics, School of Medicine, Bahcesehir University, Istanbul, Turkey; 3 Libin Cardiovascular Institute of Alberta, Department of Cardiac Sciences, University of Calgary, Calgary, Canada; 4 Center for Molecular Simulations, Department of Biological Sciences, BI-447, University of Calgary, 2500 University Drive, NW, Calgary T3A2T3, Canada

**Keywords:** hERG K channel, A_2A_ adenosine receptor, 5HT-4 receptor, Molecular docking, Molecular dynamics simulations, Drug design, Drug databases

## Abstract

**Background:**

The human ether-a-go-go related gene 1 (hERG1), which codes for a potassium ion channel, is a key element in the cardiac delayed rectified potassium current, I_Kr_, and plays an important role in the normal repolarization of the heart’s action potential. Many approved drugs have been withdrawn from the market due to their prolongation of the QT interval. Most of these drugs have high potencies for their principal targets and are often irreplaceable, thus “*rehabilitation*” studies for decreasing their high hERG1 blocking affinities, while keeping them active at the binding sites of their targets, have been proposed to enable these drugs to re-enter the market.

**Methods:**

In this proof-of-principle study, we focus on cisapride, a gastroprokinetic agent withdrawn from the market due to its high hERG1 blocking affinity. Here we tested an *a priori* strategy to predict a compound’s cardiotoxicity using *de novo* drug design with molecular docking and Molecular Dynamics (MD) simulations to generate a strategy for the rehabilitation of cisapride.

**Results:**

We focused on two key receptors, a target interaction with the (adenosine) receptor and an off-target interaction with hERG1 channels. An analysis of the fragment interactions of cisapride at human A_2A_ adenosine receptors and hERG1 central cavities helped us to identify the key chemical groups responsible for the drug activity and hERG1 blockade. A set of cisapride derivatives with reduced cardiotoxicity was then proposed using an *in-silico* two-tier approach. This set was compared against a large dataset of commercially available cisapride analogs and derivatives.

**Conclusions:**

An interaction decomposition of cisapride and cisapride derivatives allowed for the identification of key active scaffolds and functional groups that may be responsible for the unwanted blockade of hERG1.

## Background

Several classes of potassium channels are involved in regulating the heart rate by setting the amplitude and duration of the action potential and the resting membrane potential. Abnormalities in the function of these ion channels due to inherited mutations or pharmacological blockage can prolong the duration of the action potential, leading to the development of severe arrhythmias (i.e., long QT syndromes - LQTS). Genetic analysis has revealed that mutations in potassium channels, such as the human ether-a-go-go related gene (hERG) and KvLQT1, establish a molecular basis for LQTS [1-4]. A growing number of diseases have also been linked to genetic mutations in potassium channels. The channelopathies related to potassium channels include various cancer types, type 2 Bartter’s syndrome, type 1 episodic ataxia, and hyper-insulinemic hypoglycemia [4]. The best-known feature of the hERG1 channel is its unique promiscuity in binding to a wide range of organic molecules. A broad panel of organic compounds used in common cardiac and non-cardiac medications (e.g., antibiotics, antihistamines and antibacterial agents) are thought to cause a reduction in the repolarizing current I_Kr_ by blocking the central cavity of hERG and similar channels, leading to ventricular arrhythmia [5]. Several Food and Drug Administration (FDA) approved drugs (i.e., terfenadine, cisapride, astemizole and grepafloxin) have been withdrawn from the market, while others like thioridazine, haloperidol, sertindole, and pimozide have been restricted due to their effect on the function of the hERG channel. The discovery of drug-related arrhythmias has led to mandatory drug screening for hERG1 blockage by both the FDA and the European Medicines Agency (EMEA). Because most of these drugs have high binding affinity profiles for their principal targets, “*rehabilitation*” studies aimed at reducing their side effects (i.e., decreasing their high hERG1 blocking affinities) while keeping them efficient at binding to their original targets have become increasingly common. These studies may allow these drugs to reenter the market.

We chose cisapride to study as a model hERG blocker that can potentially be rehabilitated and returned back to the market. Researchers at *Janssen Pharmaceutica* discovered cisapride (a gastroprokinetic agent; trade names: prepulsid, propulsid) in 1980. Cisapride, a prokinetic agent, increases gastrointestinal motility and acts as a selective serotonin agonist for 5HT-4 receptors. It also relieves gastrointestinal symptoms (i.e., constipation and bloating) by indirectly stimulating the release of acetylcholine in muscarinic receptors. In the gastrointestinal tract, the activation of these receptors stimulates smooth muscle contraction. While cisapride’s main target is thought to be the 5-hydroxytryptamine receptor 4 (5HT-4), it can selectively target a number of other G-protein-coupled receptors (GPCRs). For example, cisapride has binding affinities for 5HT-4 (~14 nM) and adrenergic receptors (16 nM at the α-1 adrenergic receptor). Cisapride was marketed in the USA from 1993 to 2000; the use of cisapride has been associated with 341 reports of cardiovascular problems, including LQTS and torsades-de-pointes, and 80 reports of death [6]. After seven years on the market, cisapride was withdrawn in the USA and was limited for use in many other countries due to its high hERG1 blocking affinity (IC_50_ 6.5 nM, Mohammad *et al*. [7]; IC_50_ 44.5 nM, Rampe *et al*. [8,9]*;* IC_50_ 15.0 nM Drolet *et al*. [10]). A goal of our proof-of-principle study is to establish the feasibility of an *in silico* cardiotoxicity assessment with a multi-target computational approach for rehabilitation that:

(i) Assesses drugs for their hERG-blocking ability;

(ii) Identify the active components responsible for the original target activity;

(iii) Caps or modifies the moieties responsible for the hERG blockade.

**Figure 1:**
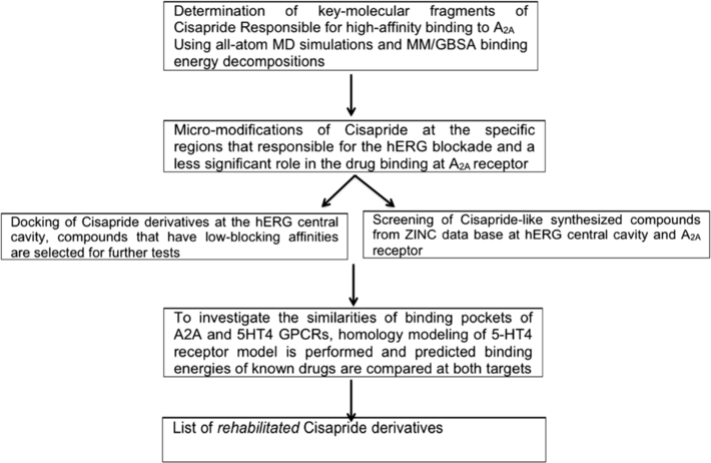
The block-scheme for the work-flow in the computational rehabilitation of cisapride derivatives.

**Figure 2:**
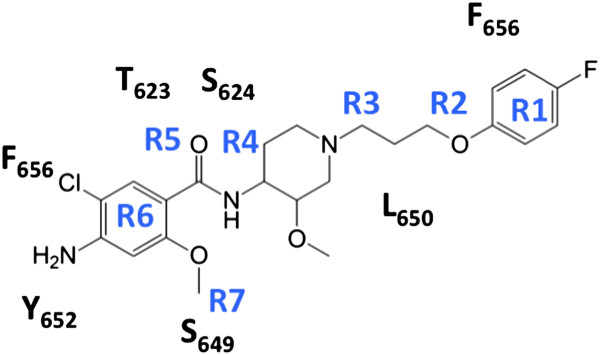
**The group definition of cisapride (i.e., R1 = -C**_**6**_**H**_**4**_**-F; R2 = -O-CH**_**2**_**-; R3 = -CH**_**2**_**-CH**_**2**_**-; R4 = -C**_**5**_**H**_**3**_**N-O-CH**_**3**_**; R5 = -NH-CO-; R6 = C**_**6**_**H**_**2**_**-Cl-NH**_**2**_**; R7 = -O-CH**_**3**_**) for per-group decomposition and inter-monomer contacts from the MM/GBSA analysis.**

The workflow chart is shown in Figure 1. We combined step-by-step ligand modification focusing on key functional groups (Figure 2), two-target receptor docking, and molecular dynamics (MD) simulations to achieve this goal. First, we determined the key-molecular fragments of cisapride responsible for its high-affinity binding to the A_2A_ receptor using all-atom MD simulations and Molecular Mechanics/Generalized Born Surface Area (MM/GBSA) binding energy decompositions, which are similar to the approaches used in previous studies [11-13]. Next, small modifications of the sites responsible for the hERG blockade that played a less significant role in the stabilization of cisapride in the binding pocket of the A_2A_ receptor were made. Our previously developed atomistic models of the open-state hERG1 channel were used to guide the modifications of cisapride. The most potent drug variants were tested for their binding to the central cavity of the hERG1 channel. The compounds that showed low affinities were then selected for further analysis. Because no crystal structure of the 5HT-4 receptor is currently available, we used a recently crystallized agonist-bound active GPCR from the same family as the target (the human adenosine A_2A_ receptor, PDB ID: 3QAK) [14]. Since cisapride can selectively bind to a number of other GPCRs (including A_2A_ receptor) with a similar range of binding affinity at a similar binding pocket, we chose to work with the available agonist-bound active target site of the A_2A_ receptor to screen cisapride and its derivatives. Prior binding site [15] and comparative homology modeling studies for these receptors indicate a high similarity between the 5HT-4 and A_2A_ binding pockets. Finally, a list of possible modifications to the original cisapride molecule was generated. MD simulations with MM/GB-SA computations, database drug screening and *de novo* design studies clearly showed that the shorter alkyl chain in cisapride analogues are key to retaining their binding to the A_2A_ receptor while remediating the blockade of the hERG1 channel. To compare *in silico* results to *de novo* developments we screened a large panel of already synthesized cisapride analogs. Small molecule databanks (i.e., ZINC [16]) were screened for synthesized cisapride derivatives and the relevant literature was reviewed to identify their activity in both the GPCR and hERG targets. Dual target docking combined with all-atom MD simulations were used to establish the key interactions between cisapride and its derivatives responsible for their high-affinity binding to the A_2A_ and how they receptor triggering hERG1 blockade. Using this combination of techniques it is possible to assess novel compounds *a priori* for their cardiotoxicity risks associated with hERG1 blockade as well as to identify sets of functional groups responsible for on-target binding.

## Methods

### Molecular docking

The structures were initially optimized using Schrodinger’s Macromodel module to perform an all-atom MM geometry optimization. The optimization was carried out with the OPLS 2005 force field and the Polak-Rebiere Conjugate Gradient (PRCG) energy minimization method with a 0.001 kcal mol^−1^ Å^−1^ energy gradient convergence criterion [17]. The resultant ligand structures were docked to the targets using the following docking algorithms: Glide/Induced Fit Docking (IFD) [18], FlexX [19], Autodock [20], and Generalized Optimized Ligand Docking (GOLD) [21]. The details of the docking algorithms are described below:

*Glide/IFD*: The Glide-XP (extra precision) (v.5.0) and Induced Fit Docking (IFD) modules of the Maestro suite were used for the docking calculations. The docking studies were performed with the following steps: (i) constrained minimization of the receptor with an root mean square deviation (RMSD) with a cutoff of 0.18 Å; (ii) initial Glide docking of each ligand using soft potentials; (iii) refinement of the derived docking poses (i.e., minimization of the docking poses within 20 Å of the ligand poses) with Schrodinger’s Prime module; and (iv) Glide re-docking of the protein-ligand complexes. *GOLD*: The GOLD program (v.5.0.1) was used with two default docking scores (GOLD Fitness and ChemScore). Part of the receptor's flexibility was accounted for by assigning flexibility to 10 specific amino acid residues at the active site. The side chains of these amino acid residues were selected as flexible rotamers. The rotamers progressed by 10° increments to cover a full 360° rotation. The default genetic algorithm parameters (100 for the population size, 5 for the number of islands, 100000 for the number of genetic operations and 2 for the niche size) were used. However, the maximum number of runs was set to 100 for each docking simulation. *FlexX:* The FlexX program (v.4.0) from BioSolveIT was also used. The default algorithm parameters were used for the docking and construction of the active sites of the receptor. The solutions per fragment and per iteration were both set to 2000. *AutoDock (v.4.0):* The number of grid points in each direction was 126 with a grid spacing of 0.4 Å. The number of hybrid Genetic Algorithm-Local Search (GA-LS) runs was 200.

### Homology modeling

The SWISS-MODEL homology modeling program [22] was used for the development of the 5HT-4 receptor model. A multiple sequence alignment was performed using the CLUSTALW algorithm [23]. A β1 adrenergic receptor (A_2A_ with a carvedilol agonist, PDB ID: 4AMJ) was used as the template because it had the highest sequence identity percentage in the sequence alignment (41%). Protein models were generated from the alignment in a stepwise manner. The backbone coordinates for the aligned positions were extracted from the template and the regions of insertions/deletions in the alignment were found by searching either a loop library or a conformational space search using constraint space programming. The templates were weighted by their sequence similarity to the target sequence and outlier atomic positions were excluded. The scoring function used for assessing favorable interactions (hydrogen bonds, disulfide bridges) and unfavorable close contacts for determining side chain conformations was derived from a backbone-dependent rotamer library [24].

### MD simulations

All-atom MD simulations were carried out using CHARMM (v. c36a2) [25]. All simulations were carried out at 323 K and 1 *atm* using periodic boundary conditions (PBC) with the NPT ensemble. The particle mesh Ewald (PME) algorithm was used for the long-range electrostatic interactions. Both the A_2A_ receptor from PDB coordinates [14] and a model of the hERG1 channel [1,2,26] were embedded into the DPPC membrane bilayer using the CHARMM-GUI membrane builder protocol [27]. Structures were minimized and equilibrated with gradually decreasing harmonic constraints (i.e., they were initialized with 10.0 and 5.0 kcal mol^−1^ Å^−2^ for the backbone and side chains, respectively, and gradually decreased to 0.5 and 0.1 kcal mol^−1^ Å^−2^, respectively) over 2 ns (for equilibration) and then subjected to a 50 ns production run.

### MM/GBSA computations

The enthalpies of cisapride binding to both targets were computed as averages from an ensemble of structures (5000 for each binding pose) sampled from evenly distributed points over five independent 50 ns runs. The electrostatic contributions to the desolvation components of the binding enthalpies were obtained by using the Generalized Born Solvation Energy Module and an Implicit Membrane (GBIM) module as implemented in CHARMM [28,29]. A dielectric constant of 2 was assigned to the protein, and the protein-solvent surface was defined using a set of optimized atomic radii from Nina *et al*. [30]. Following the numerical recipe of Chandra *et al*. [31], the membrane was represented as a 24 Å slab with a dielectric constant of 2. To better describe the lipid dynamics, we extracted the positions of the lipid’s heavy atoms in the bilayer and used them to represent the neutral slab around the protein and cisapride. The protein-ligand interaction components of the binding enthalpies were computed following a standard protocol with infinite cut-offs [12,32].

## Results and discussions

### MD simulations

**Figure 3:**
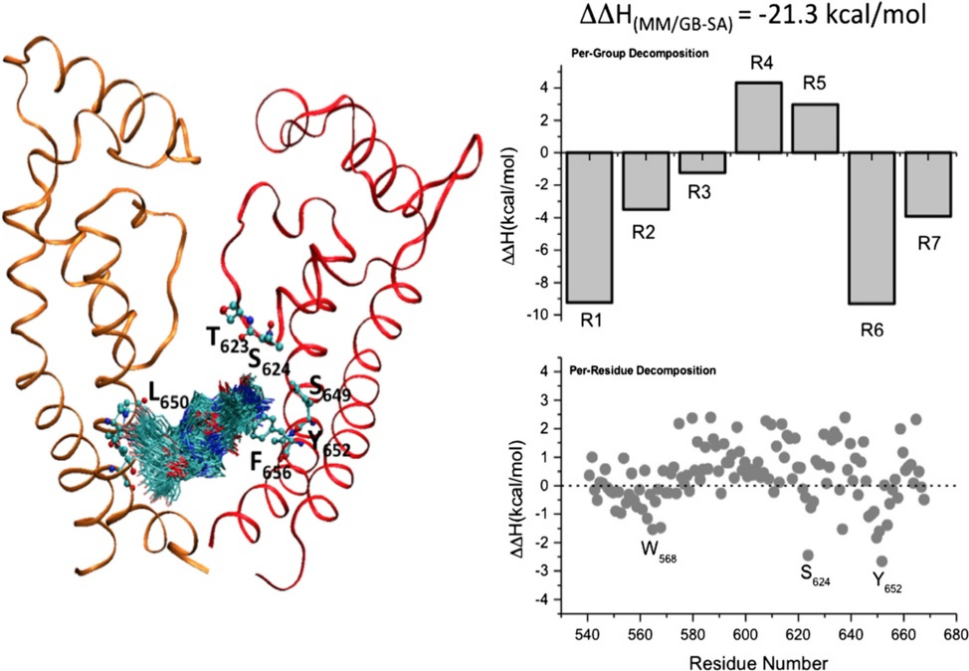
**(left) Dynamics of cisapride in the hERG1 cavity (Backbone RMSD after 50 ns ~5.0 Å for a tetramer, 4.2 Å for a monomer; the pore domain has an RMSD of ~2.9 Å).** (right) Summary of the MM/GB-SA decomposition analysis (per-residue and per-group contributions) from the last 40 ns of the MD simulation of the hERG1-cisapride complex. (see Figure 2 for a definition of the R groups at cisapride).

**Figure 4:**
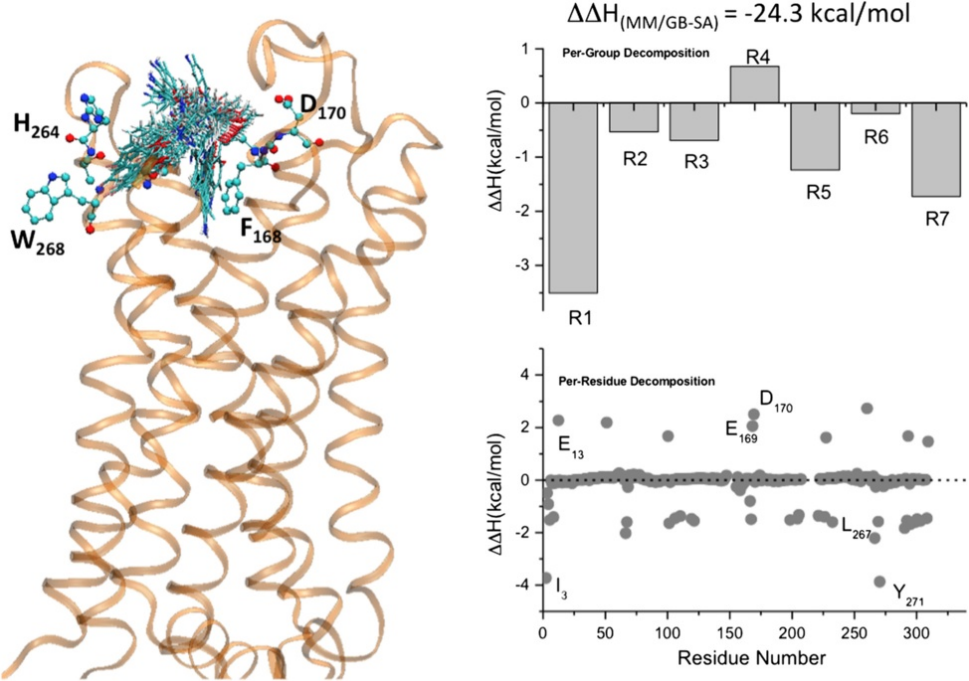
**(left) The dynamics of cisapride in the A**_**2A **_**binding pocket (Backbone RMSD after 50 ns ~3.1 Å).** (right) Summary of the MM/GB-SA decomposition analysis (per-residue and per-group contributions) from the last 40 ns of the MD simulation of the A_2A_-cisapride complex.

**Figure 5:**
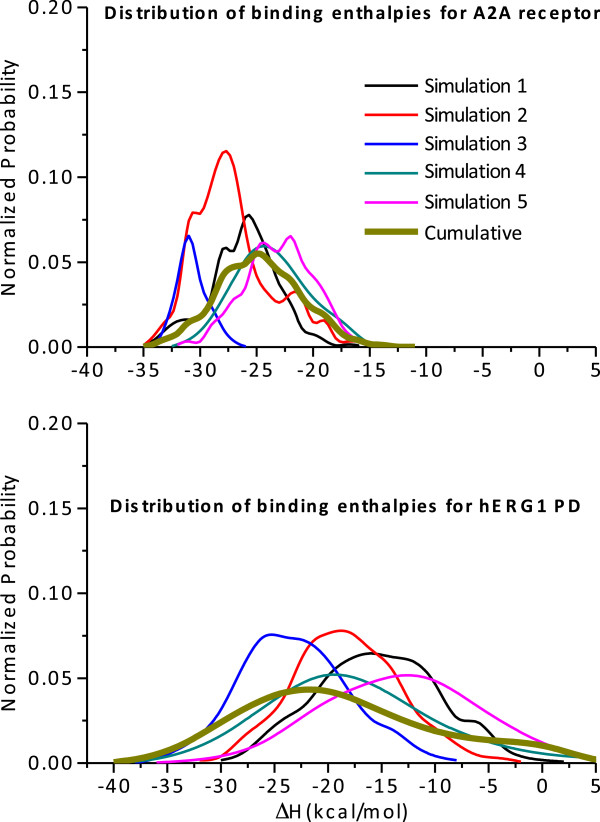
Normalized probability distribution function of the binding enthalpies for cisapride to WT-A2A and WT-hERG1 proteins.

As stated in the Introduction, one of the major goals of this study was to identify the determinants of the cisapride blockade of hERG and its binding to the A_2A_ receptor which serves as a suitable model for the 5HT-4 receptor. Although our docking studies unambiguously identified binding pockets in both proteins, the orientation of the bound ligand was less well defined. To account for the limited resolution of our docking studies, we ran five independent 50 ns full-membrane all-atom molecular dynamics (MD) simulations using the top 5 poses from docking analysis as a starting point. All simulations were found to produce stable trajectories with membrane proteins displaying heavy atom RMSD values of ~3 to 4 Å, which is comparable to root mean squared fluctuation (RMSF) estimates from previous studies of K-channels with available crystal structures [33]. The averaged locations of the ligand after MD simulations are shown in Figures 3 and 4. Cisapride bound in either membrane protein (A_2A_ receptor or hERG1 open-state channel model) displays significant conformational dynamics in the binding site with an average RMSD value of ~1.1 Å relative to the average structure. The backbone RMSD of the hERG tetramer channel after 50 ns was approximately 5.3 Å including deviations from the symmetric tetramer; the corresponding values for the monomers were from 3.5 to 4.2 Å. The positional fluctuations in backbone atoms of residues forming the pore domain (PD) of the hERG1 monomer plateaued at a RMSD of ~2.4-2.9 Å. These values are similar to those reported previously for MD simulations of Kv channels [32]. The backbone RMSDs of the A_2A_ receptor plateaued at ~3.1 Å. The considerable dynamics of the receptor and the bound ligand suggest that explicitly accounting for site and ligand flexibility in evaluation of the binding energies is necessary. To circumvent obvious limitations of the chosen docking strategy and to identify key interacting partners e.g. relevant amino-acid residues and key functional groups in the drug, we performed MM/GBSA computations to estimate the binding affinity of cisapride in the A_2A_ receptor and the open-state hERG1 model. The distribution of binding enthalpies from 5 independent simulations is shown in the Figure 5. The average binding enthalpies for cisapride to the hERG1 and A_2A_ are –21.3 ± 2.8 kcal/mol and -24.3 ± 1.9 kcal/mol, respectively.

Analysis of the per-residue and per-functional group contribution to binding enthalpies allows us to determine residues and drug fragments, which uniquely determines the binding mode of cisapride in both systems. (Figures 3 and 4) The key residues that determine the thermodynamics of cisapride stabilization in the hERG1 cavity are S624 from the pore helix, Y652 and F656 from the distal S6 and, surprisingly, W568 from the S5 helix. The high-affinity intra-cavity blockade of hERG1 by cisapride depends on the hydrogen bonds formed by the pore-helix and on stabilizing aromatic interactions with residues in the S5 and S6 helices. The drug dimensions allow for inter-subunit interactions (see Figure 2) with matching hydrophobic moieties in the hERG1 cavity while the carbonyl backbone of cisapride interacts strongly with T623 and S624. The binding mode is similar to that for dofetilide and other molecules known to restrict hERG1 K^+^ currents by binding to the protein’s internal cavity [3,26]. Most of the residues were mapped in previous experimental studies and are thought to be responsible for high-affinity blockades [34-38]. Importantly for both systems, docking studies were able to identify key amino acids and functional groups and thus results obtained from average protein structure and flexible ligand docking appear to be sufficiently predictive to allow for micromanagement of the drug.

The decomposition of binding enthalpies for cisapride binding to the A_2A_ adenosine receptor is illustrated in Figure 4. While there is a cluster of acidic residues involved in drug binding (E13, E169 and D170), the desolvation penalty for the negative charges located on the surface of the receptor counter balances favorable charge interactions and contributes unfavorably overall to drug stabilization in the pocket. Instead, the bound cisapride is stabilized to a large extent by a network of aromatic and amphipathic groups lining the binding pocket. The constellation of residues includes Y271, L267, I252, T256 and M270 that interact with the linker and the rings (R1, R3, and R6). (See Figure X for labeling of the regions of cisapride) The binding of cisapride is further stabilized by interactions between the residues S67, T68 and F168, and the moieties of the R2 and R3 regions. It is worth mentioning that all of these residues are involved in the formation of binding pockets for adenosine and other agonists, according to previous studies and recently published crystal structures [14]. F168 forms binding interactions with the central methoxy groups of the bound molecule, and other key residues (S67, T68 and Y271) interact with amphipathic functional groups on aromatic rings. The molecular fragments of the drug that form stable interactions with the residues in the A_2A_ receptor are as follows: a methoxy group on the central heterocyclic ring (R4), a terminal aromatic ring (R1), and a methoxy group (R7) on the benzamide ring. Interestingly, it has been shown that modification of the benzamide ring is essential for modulation of the activity of cisapride derivatives [39].

To better understand the effects of each fragment of cisapride on the A_2A_ adenosine receptor binding and blocking affinities of hERG1, we also performed per-group decomposition of the binding enthalpies for drug binding to hERG1. The cisapride fragments were defined according to the topology definition of the CHARMM general force field [25,40] and are illustrated in Figure 2. The resulting decomposition is shown in Figures 3 and 4, allowing us to focus on targeted modifications of the fragments. It is apparent that to inhibit binding to hERG1, one may need to modify one or both of the terminal rings (R1, R6) as well as the flexible linkers (R2, R3). Cisapride bound to the hERG1 cavity interacts with a ring of hydrophobic and aromatic residues (Y652, A653 and F656) and the Ser/Thr-rich apex of the pore helix (T623, S624) [2]. Experimental studies have also demonstrated that the matching interactions between cisapride and aromatic rings (F656 and Y652) are essential for the hERG blockade [41]. The decomposition analysis shows that one of the key interactions important for the hERG blockade but unrelated to the high-affinity binding of cisapride to the A_2A_ receptor is the van-der-Waals interactions between the long alkyl chain linker of the drug and a number of hydrophobic residues in the hERG cavity. The docking/MD simulations strategy adopted in this study appears to have yielded a potential route to rehabilitate cisapride. In our next step, we attempt to remove fragments responsible for drug stabilization in the inner-cavity of hERG1 channel, while retaining its binding to the A_2A_ receptor. Several parallel approaches were considered for *de novo* development of non-blocking cisapride collected in Table 1:

**Table 1:** **Selected cisapride analogues from the *de Novo *** **drug design study**

**Ligands**	**2D structures**	**A**_**2A **_**receptor docking score (kcal/mole)**	**hERG1 docking score (kcal/mole)**
**Cisapride**		-8.48	-7.80
**Cisapride_Frag_337**		-9.46	-4.73
**Cisapride_Frag_182**		-8.66	-5.34

The 10700 small organic molecules fragment database of Schrodinger was used to create new derivatives of cisapride (the place for replacement was previously found by a trajectory analysis of the MD simulations). Molecules were initially docked using virtual high‒throughput screening (VHTS) with subsequent Glide/XP docking for the promising derivatives (~500 compounds). Finally, the inhibitory profiles of these derivatives at hERG1 were tested using Induced Fit Docking.

(i) In the first approach, we simply targeted heteroatoms in the functional groups of cisapride molecule. They were exchanged with hydrogen (-H) to observe the effect of each of these fragments on the docking scores for both receptors.

(ii) Next, the number of –CH_2_ groups of the linker was modified to produce truncated and extended versions of the linker. This affects the distance between the terminal aromatic ring and the six-membered heterocyclic ring piperidine, thus affecting torsional energy. The length of the alkyl chain also modulates the interactions between the aromatic moieties of the drug and the Y652/F656 residues that are key components of the high-affinity blockade of hERG1.

(iii) In the last approach, the functional groups were removed based on the per-fragment decomposition, as shown in Figures 1 and 3.

A library of 10,700 molecules from the small organic molecules fragment database of Maestro was used to create new analogues of cisapride guided by energy decomposition analysis as well as the per-residue interaction energy analysis described above. The summary of the modification strategy is shown in Additional file 1: Tables S1 to S2. All of the developed molecules were initially docked using virtual high throughput screening (VHTS) followed by Glide extra precision (XP) docking for the derivatives showing low hERG blocking binding scores and high binding scores for the A_2A_ adenosine receptor (~500 compounds).

### General note on molecular docking and *De novo* drug design

**Figure 6:**
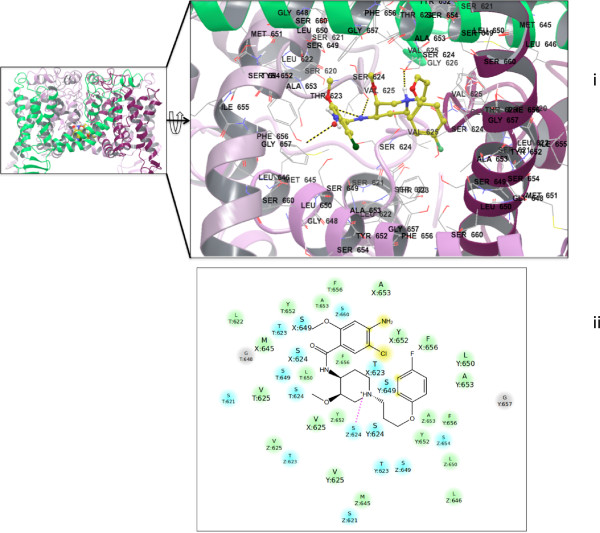
**Ligand Binding to hERG1 pore domain. (i)** The top-docking pose of cisapride in the hERG1 pore domain. (left) Binding interactions are zoomed and detailed (right). **(ii)** A 2D-ligand interaction map for cisapride binding to the hERG cavity.

**Figure 7:**
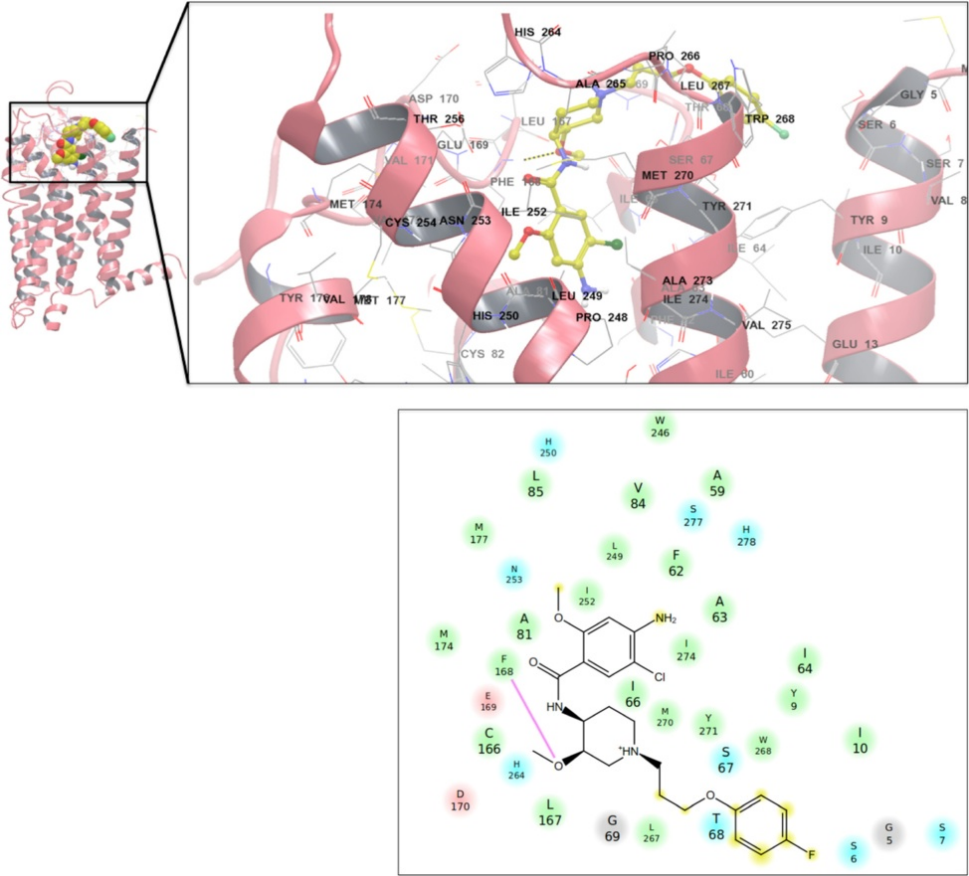
**Ligand Binding to A2A receptor.** Top panel: The top-docking pose of cisapride in the human A_2A_ adenosine receptor. (left) Binding interactions are zoomed and detailed (right). Bottom panel: A 2D-ligand interaction map for cisapride binding to the A_2A_ receptor.

All of the derived cisapride analogues were docked to the A_2A_ (PDB ID: 3QAK) active site using the approach described in the methods section. They were also docked to the pore domain of hERG1 model, and the cisapride derivative results were compared to the original cisapride docking scores. (Figures 6 and 7, and Additional file 1: Table S1) Four different molecular docking programs (AutoDock, Glide, FlexX, and GOLD) were used to study the binding interactions of cisapride and the cisapride derivatives in the A_2A_ adenosine receptor and the hERG1 central cavity. Since the FlexX and AutoDock docking programs both underestimated the interaction energies of cisapride and its derivatives at both targets (i.e., hERG and A_2A_), GOLD and Glide/XP are generally preferred for the evaluation of binding affinities of known compounds. Our previously developed model of the trans-membrane domains, S1-S6, of open-state hERG1 [1,26] was used to model the binding interactions of cisapride and its derivatives in the pore domain of the channel. Initially, cisapride was docked to A_2A_ receptor as well as hERG1 model, and these binding scores were used as threshold values for all four docking programs. Cisapride derivatives that displayed similar/higher binding scores (absolute values) in the A_2A_ receptor and lower binding scores in the hERG1 pore domain were considered for further analysis. For example, one of them (#11) labeled as Cisapride-D11 in the text had docking scores similar to original cisapride in the A_2A_ binding pocket and considerably lower docking scores in the hERG PD, thus, it was considered for further rehabilitation.

### Effect of functional group substitution on drug binding to A_2A_ receptor and hERG1 channel

All of the candidates with hydrogen substitutions by heteroatoms (i.e., Cisapride-D1 and Cisapride-D8 in the Additional file 1: Table S1) had docking scores similar to those of cisapride binding to the A_2A_ receptor. However all of these derivatives retained high docking scores for binding to the hERG PD and therefore were discarded. Some of the new derivatives, such as Cisapride-D2 (an exchange of a –Cl group with –H), led to a decrease in the docking score in the A_2A_ receptor (i.e., the Glide/XP docking score decreased from -8.48 to -5.07 kcal/mole for Cisapride-D2), thus these derivatives were also discarded. (Additional file 1: Table S1) While the removal of some functional groups from cisapride did not significantly reduce the docking scores of compounds at A_2A_, their hERG PD bindings were still too high (i.e., Cisapride-D4, Cisapride-D5).

Varying the number of –CH_2_ groups in the linker, R3 between the terminal aromatic ring, R1, and the central heterocyclic ring, R4, affected the docking scores of compounds in the hERG PD. (See Figure 2 for labeling) While decreasing the number of -CH_2_ groups from three (original cisapride) to one reduced the docking score of the derivative in the hERG1 central cavity, (i.e., for Cisapride-D11, GOLD docking score went from -9.42 to -7.40 kcal/mole; the Glide/XP docking score went from -7.80 to -7.09 kcal/mole) increasing the number of –CH_2_ groups from 3 to 4 did not significantly affect the docking score of the derivative in the hERG1 PD (i.e., for Cisapride-D9, the GOLD docking score went from -9.42 to -9.03 kcal/mole; the Glide/XP docking score went from -7.80 to -7.45 kcal/mole) (Additional file 1: Table S1). Therefor for further rehabilitation studies, only a decreased number of –CH_2_ groups in the cisapride backbone (i.e., Cisapride-D11) was considered. Derivatives of Cisapride-D11 were generated using the Combinatorial Library Enumeration and Screening module of the Maestro molecular modeling package and the corresponding docking results were compared with that of original Cisapride-D11 (Additional file 1: Table S2). For this purpose, approximately 600 default fragments in Schrodinger’s Enumerate module were used, and 8600 new cisapride derivatives were generated. After the derivative generation, the compounds were prepared (protonation states were determined at a pH of 7) with the LigPrep module of Maestro and energy minimization was performed for the structures using the PRCG energy minimization method. Structures were then docked onto the A_2A_ adenosine receptor via the Glide High Throughput Virtual Screening method (Glide/HTVS). This was used to reduce the large number of derivatives to a manageable number for further analysis. The top 100 compounds according to the Glide/HTVS docking scores were used for Glide extra precision (XP) docking both in the A_2A_ active site and the hERG1 central cavity. The docking results of the selected derivatives of Cisapride-D11 are tabulated in the Additional file 1: Table S2. One of the major outcomes of this *in silico* study is that simple truncation of the flexible linker appears to be sufficient to remediate unwanted off-target interactions of cisapride. Obviously, it is not sufficient just to claim that such modifications will lead to a potent drug; it requires verification. One of the reasons for choosing cisapride is the availability of a large number of previously evaluated cisapride derivatives. Accordingly, to test similarities and differences in predicted cardiotoxicity (hERG1 blockade) and efficacy of the cisapride derivatives we used the ZINC database and literature mining.

### Comparison study with a database of available drug databases

94 cisapride derivatives (with >80% structural similarity with cisapride) taken from the ZINC databank were used to obtain the docking scores/poses for binding to the human adenosine A_2A_ receptor and the hERG1 pore domain using the Glide/XP docking program. The compounds that had docking scores (absolute values) greater than -7.00 kcal/mole in the A_2A_ receptor binding site (28 cisapride derivatives) were evaluated for their docking scores in the hERG PD (Additional file 1: Tables S3 and S4). Results showed that a few promising compounds (i.e., ZINC05998832, ZINC58529167, ZINC13834042, ZINC20621758, ZINC43023913) with docking scores less than (absolute values) -6.00 kcal/mole in the hERG1 PD, and greater than (absolute values) -7.00 kcal/mole in the binding pocket of the A_2A_ receptor. (Table 2)

**Table 2:** **Cisapride derivatives from the ZINC database that show low binding scores in the hERG1 PD and high binding scores in the A**_**2A **_**adenosine receptor**

**Cisapride derivatives**	**2D structure**	**Glide XP docking score @A**_**2A **_**(kcal/mol)**	**Glide XP docking score @hERG1 PD (kcal/mol)**
**ZINC05998832**		-9.45	-4.62
**ZINC58529167**		-7.57	-4.51
**ZINC13834042**		-7.39	-5.51
**ZINC20621758**		-7.22	-5.67
**ZINC43023913**		-7.21	-5.01

**Figure 8:**
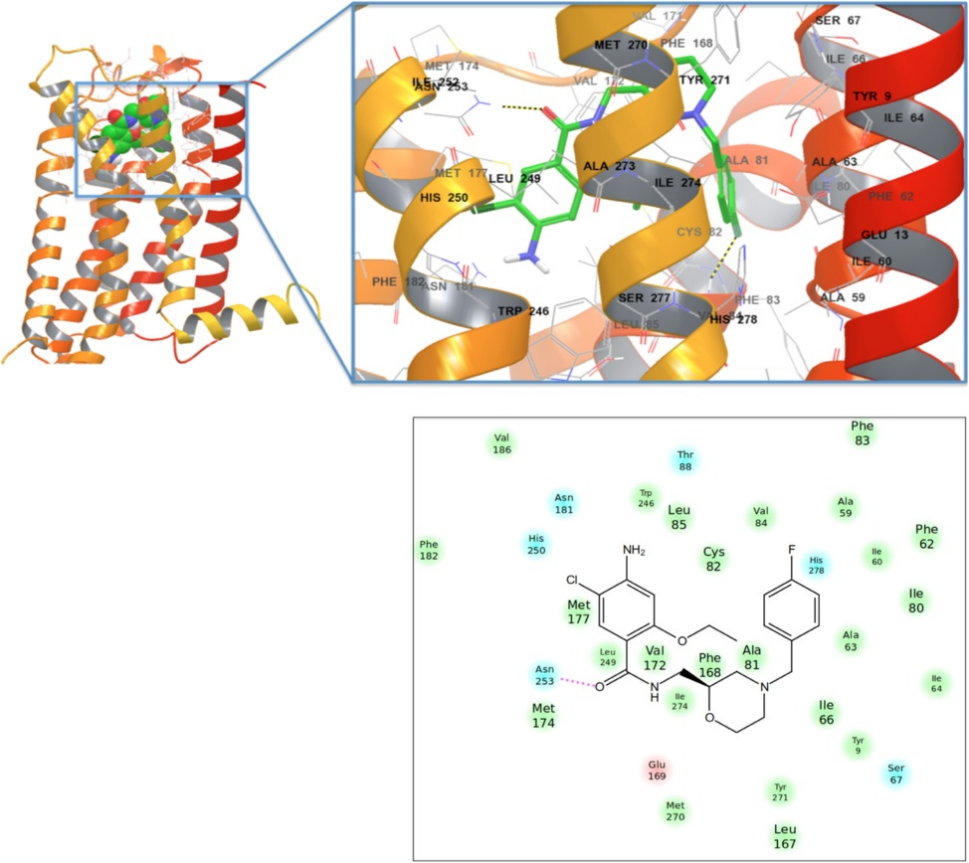
**Mosapride bidning to hERG1 pore domain.** Top panel: The top-docking pose of mosapride that shows a low hERG PD blocking affinity in the human A_2A_ adenosine receptor. (left) Binding interactions are zoomed and detailed (right). Bottom panel: 2D-ligand interaction map for mosapride binding to the A_2A_ receptor.

Interestingly, ZINC20621758, also known commercially as mosapride, was detected via blinded dual-target screening to be a safer 5HT-4 agonist than cisapride. Potet *et al*. [6] showed that mosapride does not block the hERG channel, which is in agreement with lower binding scores predicted *in silico*. It is also a well-known agonist of the 5HT-4 receptor. The IC_50_ of mosapride binding to the hERG channel is ~16.5 μM. Mosapride carries a significant decrease of cardiovascular risks related to alterations in QT intervals according to preclinical studies [42,39,43]. Carlsson *et al*. used a rabbit model of the acquired long QT syndrome, and while cisapride prolonged the QT interval, mosapride did not [39,44,45]. One of the key differences between mosapride and cisapride is a shorter linker between aromatic rings that, as predicted in the dual-target *de novo* drug modeling, will have an immediate effect on the drug-hERG PD interactions. Figure 8 shows the top Induced Fit docking pose of mosapride in the A_2A_ receptor. Cisapride and mosapride have different binding modes in the A_2A_ receptor due to a different number of CH_2_ groups in the linker. Additional file 1: Figure S2 shows a superposition of the top-docking poses of cisapride and mosapride.

The other ZINC compounds in Table 2 also exhibit high docking scores in the A_2A_ receptor; however there are no experimental results available in the literature for these compounds. However, ZINC05998832 (*aka* fluoroclebopride) is a close analog of clebopride. The reported IC_50_ of clebopride in the hERG PD is 0.62 μM. Clebopride is commercially available in Spain and Italy as a gastroprokinetic drug. Although its cardiotoxicity has not been reported in clinical studies, *in vitro* studies have shown that clebopride prolongs the cardiac action potential duration at 90% (but not 50%) repolarization at 10 μM [44]. Tack *et al.*[44] also found that no cardiovascular safety concerns were reported for the newer selective 5HT-4 agonists prucalopride, velusetrag, and naronapride or for the non-selective 5HT-4 agonists with no hERG or 5HT-1 affinity, such as renzapride, clebopride, and mosapride. Importantly, the experimental data available for several ZINC compounds indicated nM affinity against 5HT-4 and 5HT-2 receptors, corresponding to high affinity binding for several of the compounds studied (ZINC20621758, Ki ~113 nM; ZINC43023913, 79 nM; ZINC13834042, 2250 nM and ZINC05998832 ~ 283 nM, respectively), all with very low docking scores in the hERG PD. Although a few cisapride analogues (such as ZINC28087327, Ki 40.6 nM in 5HT-4; ZINC43024452, Ki 3.16 nM in 5HT-4) were correctly predicted by *in silico* screening to have high experimental binding affinities in the 5HT-4 receptor, they also had high docking scores in the hERG PD (Additional file 1: Tables S2 and S3).

### On the transferability of A_2A_ predictions to 5HT-4 receptors

**Figure 9:**
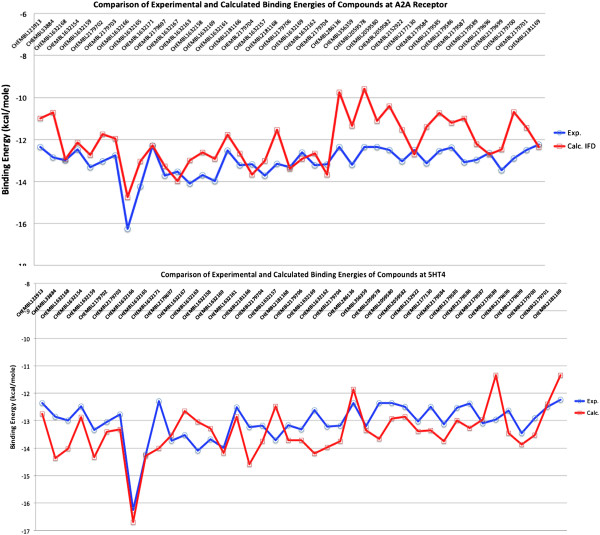
**Comparison of experimental and calculated (IFD docking scores) binding energies of compounds.** Top panel: The A2A receptor and Bottom panel: The 5HT-4 receptor. (Ki values are converted to kcal/mole for comparison, T = 300 K, R = 8.314 J/molK; If a compound has different Ki values for same target, the average of these values is used).

There is a well-known challenge in relating the top binding poses produced by docking to the most stable ligand orientations in the binding pocket. Many organic molecules are flexible with a number of rotating bonds. The previous section on mapping the relevant binding sites in the receptor suggests that some of these residues are also responsible for selective substrate and agonist binding to human 5HT-4 [15,46]. It is worth mentioning that the main target of cisapride in gastrointestinal tissues is not the A_2A_ receptor but the 5HT-4 receptor. To investigate the similarities of the binding pockets of two GPCRs, namely, 5HT-4 and A_2A_ receptors, we performed homology modeling with subsequent drug docking. The homology modeling was performed using SWISS-MODEL for 5HT-4 (using the highest sequence identity scores of a multiple sequence alignment). β1 adrenergic receptors (with agonist bound) resulted in the highest sequence identity percentage from a sequence alignment (41%) and it was used as a template. Superposition of the 5HT-4 model protein and the A_2A_ crystal structure reconfirmed that there is high structural similarity between these two GPCRs (RMSD of 2.4 Å) (Additional file 1: Figure S1). Furthermore, most of the residues identified by MD simulations as essential for drug stabilization are well-known determinants of high-affinity binding in the 5HT-4 system. We also used the ChEMBL database to search for compounds targeting the 5HT-4 receptor (CHEMBL1875). ChEMBL is a database for bioactive drug-like small molecules that contains abstracted experimental bioactivities from the scientific literature (i.e., binding constants and pharmacology and ADME/Tox data) [34,35]. In total, 419 associated bioactivity results for the 5HT-4 receptor were found. The results were ranked based on Ki values, and compounds that had better Ki values than 1 nM in the 5HT-4 receptor were kept for consideration. Our aim was to test the docking score accuracies for compounds in the binding pockets of the A_2A_ and 5HT-4 receptors. The protonation states were determined at pH 7, and structures were optimized using the LigPrep module of Maestro. Subsequently, these compounds were docked to the A_2A_ and 5HT-4 receptors using Glide/XP Induced Fit Docking. Considering the simplicity of the docking approach, our results show a high correlation between the experimental and predicted binding energies (r^2^ > 0.6 for both compounds in the A_2A_ and 5HT-4 receptors, docking results, Figure 9). Structures with bioactivity results in the 5HT-4 receptor were also docked in the hERG PD using Induced Fit Docking. Compounds with low predicted binding affinities in the hERG PD are listed in Additional file 1: Table S5 and can guide future studies. Our findings reconfirm the similarity of the binding pockets of the A_2A_ and 5HT-4 receptors as well as the transferability of our results.

## Conclusions

A cross analysis of the interactions between cisapride and its analogues with the human A_2A_ adenosine receptor and the hERG1 central cavity led us to formulate a computational approach to the rehabilitation of drugs withdrawn from the market due to pro-arrhythmic activity. Our SAR study of cisapride and its derivatives allows for the *in silico* evaluation of drug potency and the drug’s ability to block hERG. Molecular docking results using both *de novo* designed compounds and available cisapride derivatives in the ZINC drug database showed that the shorter alkyl chains in the cisapride analogues is key element to retaining their binding to the A_2A_ receptor and remediating the blockade of the hERG1 channel. We believe that a simple dual-target ‘*rehabilitation*’ strategy based on an optimization against two protein target structures may be applied to other drugs withdrawn from the market due to their side effects, and may lead to the reuse of these drugs.

## Competing interest

The authors declare that they have no competing interests.

## Authors’ contributions

SD performed modeling, created databases, analyzed results and wrote the manuscript. TR performed molecular modeling, performed database search, organized results and wrote the manuscript. HJD conceived idea of this study, identified target molecules and wrote the manuscript. AC wrote the manuscript and analyzed data. SN performed free energy simulations, analyzed data and wrote the manuscript. All authors read and approved the final manuscript.

## Pre-publication history

The pre-publication history for this paper can be accessed here:

http://www.biomedcentral.com/2050-6511/15/14/prepub

## Supplementary Material

Additional file 1 Summary of Docking, De Novo Design and Modeling studies for cisapride analogues investigated. Click here for file

## References

[B1] Durdagi S Deshpande S Duff HJ Noskov SY Modeling of open, closed, and open-inactivated states of the hERG1 channel: structural mechanisms of the state-dependent drug binding J Chem Inf Model 2012 52 10 2760 2774 10.1021/ci300353u 22989185

[B2] Durdagi S Duff HJ Noskov SY Combined receptor and ligand-based approach to the universal pharmacophore model development for studies of drug blockade to the hERG1 pore domain J Chem Inf Model 2011 51 2 463 474 10.1021/ci100409y 21241063

[B3] Durdagi S Subbotina J Lees-Miller J Guo J Duff HJ Noskov SY Insights into the molecular mechanism of hERG1 channel activation and blockade by drugs Curr Med Chem 2010 17 30 3514 3532 10.2174/092986710792927886 20738248

[B4] Shieh CC Coghlan M Sullivan JP Gopalakrishnan M Potassium channels: Molecular defects, diseases, and therapeutic opportunities Pharmacol Rev 2000 52 4 557 593 11121510

[B5] Ashcroft FM Ion Channels and Disease: Channelopathies 2000 San Diego: Academic Press

[B6] Potet F Bouyssou T Escande D Baro I Gastrointestinal prokinetic drugs have different affinity for the human cardiac human ether-a-gogo K + channel J Pharmacol Exp Ther 2001 299 3 1007 1012 11714889

[B7] Mohammad S Zhou ZF Gong QM January CT Blockage of the HERG human cardiac K + channel by the gastrointestinal prokinetic agent cisapride Am J Physiol-Heart C 1997 273 5 H2534 H2538 10.1152/ajpheart.1997.273.5.H2534 9374794

[B8] Rampe D Roy ML Dennis A Brown AM A mechanism for the proarrhythmic effects of cisapride (Propulsid): high affinity blockade of the human cardiac potassium channel HERG Febs Lett 1997 417 1 28 32 10.1016/S0014-5793(97)01249-0 9395068

[B9] Walker BD Singleton CB Bursill JA Wyse KR Valenzuela SM Qiu MR Breit SN Campbell TJ Inhibition of the human ether-a-go-go-related gene (HERG) potassium channel by cisapride: affinity for open and inactivated states Brit J Pharmacol 1999 128 2 444 450 10.1038/sj.bjp.0702774 10510456 PMC1571630

[B10] Drolet B Khalifa M Daleau P Hamelin BA Turgeon J Block of the rapid component of the delayed rectifier potassium current by the prokinetic agent cisapride underlies drug-related lengthening of the QT interval Circulation 1998 97 2 204 210 10.1161/01.CIR.97.2.204 9445174

[B11] Swanson JMJ Henchman RH McCammon JA Revisiting free energy calculations: a theoretical connection to MM/PBSA and direct calculation of the association free energy Biophys J 2004 86 1 67 74 10.1016/S0006-3495(04)74084-9 14695250 PMC1303837

[B12] Noskov SY Lim C Free energy decomposition of protein-protein interactions Biophys J 2001 81 2 737 750 10.1016/S0006-3495(01)75738-4 11463622 PMC1301550

[B13] Wright JD Noskov SY Lim C Factors governing loss and rescue of DNA binding upon single and double mutations in the p53 core domain Nucleic Acids Res 2002 30 7 1563 1574 10.1093/nar/30.7.1563 11917017 PMC101848

[B14] Xu F Wu H Katritch V Han GW Jacobson KA Gao ZG Cherezov V Stevens RC Structure of an agonist-bound human A2A adenosine receptor Science 2011 332 6027 322 327 10.1126/science.1202793 21393508 PMC3086811

[B15] Mialet J Dahmoune Y Lezoualc’h F Berque-Bestel I Eftekhari P Hoebeke J Sicsic S Langlois M Fischmeister R Exploration of the ligand binding site of the human 5-HT4 receptor by site-directed mutagenesis and molecular modeling Brit J Pharmacol 2000 130 3 527 538 10.1038/sj.bjp.0703356 10821780 PMC1572113

[B16] Irwin JJ Sterling T Musinger MM Bolstad ES Coleman RG ZINC: a free tool to discover chemistry for biology J Chem Inf Model 2012 52 1757 1768 10.1021/ci3001277 22587354 PMC3402020

[B17] Software Package Schrödinger Release 2014-1: Maestro, version 9.7 2014 New York, NY: Schrödinger, LLC

[B18] Friesner RA Murphy RB Repasky MP Frye LL Greenwood JR Halgren TA Sanschagrin PC Mainz DT Extra precision glide: docking and scoring incorporating a model of hydrophobic enclosure for protein-ligand complexes J Med Chem 2006 49 21 6177 6196 10.1021/jm051256o 17034125

[B19] Bohm HJ The development of a simple empirical scoring function to estimate the binding constant for a protein-ligand complex of known three-dimensional structure J Comput Aided Mol Des 1994 8 3 243 256 10.1007/BF00126743 7964925

[B20] Morris GM Huey R Lindstrom W Sanner MF Belew RK Goodsell DS Olson AJ AutoDock4 and AutoDockTools4: Automated docking with selective receptor flexibility J Comput Chem 2009 30 16 2785 2791 10.1002/jcc.21256 19399780 PMC2760638

[B21] Jones G Willett P Glen RC Leach AR Taylor R Development and validation of a genetic algorithm for flexible docking J Mol Biol 1997 267 3 727 748 10.1006/jmbi.1996.0897 9126849

[B22] Kopp J Schwede T The SWISS-MODEL Repository of annotated three-dimensional protein structure homology models Nucleic Acids Res 2004 32 D230 D234 10.1093/nar/gkh008 14681401 PMC308743

[B23] Thompson JD Higgins DG Gibson TJ Clustal-W - improving the sensitivity of progressive multiple sequence alignment through sequence weighting, position-specific Gap penalties and weight matrix choice Nucleic Acids Res 1994 22 22 4673 4680 10.1093/nar/22.22.4673 7984417 PMC308517

[B24] Benkert P Biasini M Schwede T Toward the estimation of the absolute quality of individual protein structure models Bioinformatics 2011 27 3 343 350 10.1093/bioinformatics/btq662 21134891 PMC3031035

[B25] Brooks BR Brooks CL 3rd Mackerell AD Jr Nilsson L Petrella RJ Roux B Won Y Archontis G Bartels C Boresch S Caflisch A Caves L Cui Q Dinner AR Feig M Fischer S Gao J Hodoscek M Im W Kuczera K Lazaridis T Ma J Ovchinnikov V Paci E Pastor RW Post CB Pu JZ Schaefer M Tidor B Venable RM CHARMM: the biomolecular simulation program J Comput Chem 2009 30 10 1545 1614 10.1002/jcc.21287 19444816 PMC2810661

[B26] Subbotina J Yarov-Yarovoy V Lees-Miller J Durdagi S Guo JQ Duff HJ Noskov SY Structural refinement of the hERG1 pore and voltage-sensing domains with ROSETTA-membrane and molecular dynamics simulations Proteins 2010 78 14 2922 2934 10.1002/prot.22815 20740484 PMC2939218

[B27] Jo S Lim JB Klauda JB Im W CHARMM-GUI membrane builder for mixed bilayers and its application to yeast membranes Biophys J 2009 97 1 50 58 10.1016/j.bpj.2009.04.013 19580743 PMC2711372

[B28] Dominy BN Brooks CL Development of a generalized born model parametrization for proteins and nucleic acids J Phys Chem B 1999 103 18 3765 3773 10.1021/jp984440c

[B29] Dominy BN Brooks CL Methodology for protein-ligand binding studies: Application to a model for drug resistance, the HIV/FIV protease system Proteins-Structure Function and Genetics 1999 36 3 318 331 10.1002/(sici)1097-0134(19990815)36:3<318::aid-prot6>3.0.co;2-k 10409825

[B30] Nina M Beglov D Roux B Atomic radii for continuum electrostatics calculations based on molecular dynamics free energy simulations J Phys Chem B 1997 101 26 5239 5248 10.1021/jp970736r

[B31] Chanda B Asamoah OK Blunck R Roux B Bezanilla F Gating charge displacement in voltage-gated ion channels involves limited transmembrane movement Nature 2005 436 7052 852 856 10.1038/nature03888 16094369

[B32] Im W Feig M Brooks CL An implicit membrane generalized born theory for the study of structure, stability, and interactions of membrane proteins Biophys J 2003 85 5 2900 2918 10.1016/S0006-3495(03)74712-2 14581194 PMC1303570

[B33] Noskov SY Berneche S Roux B Control of ion selectivity in potassium channels by electrostatic and dynamic properties of carbonyl ligands Nature 2004 431 7010 830 834 10.1038/nature02943 15483608

[B34] Vandenberg JI Perry MD Perrin MJ Mann SA Ke Y Hill AP hERG K+ chanels: Structure, Function and Clinical Significance Physiol Rev 2012 92 3 1393 1478 10.1152/physrev.00036.2011 22988594

[B35] Ng CA Torres AM Pages G Kuchel PW Vandenberg JI Insights into hERG K + channel structure and function from NMR studies Eur Biophys J Biophy 2013 42 1 71 79 10.1007/s00249-012-0808-6 22552870

[B36] Perry M Sanguinetti M Mitcheson J Revealing the structural basis of action of hERG potassium channel activators and blockers J Physiol-London 2010 588 17 3157 3167 10.1113/jphysiol.2010.194670 20643767 PMC2976011

[B37] Perry M de Groot MJ Helliwell R Leishman D Tristani-Firouzi M Sanguinetti MC Mitcheson J Structural determinants of HERG channel block by clofilium and ibutilide Mol Pharmacol 2004 66 2 240 249 10.1124/mol.104.000117 15266014

[B38] Lees-Miller JP Subbotina JO Guo JQ Yarov-Yarovoy V Noskov SY Duff HJ Interactions of H562 in the S5 Helix with T618 and S621 in the Pore Helix Are Important Determinants of hERG1 Potassium Channel Structure and Function Biophys J 2009 96 9 3600 3610 10.1016/j.bpj.2009.01.028 19413965 PMC2711401

[B39] Carlsson L Amos GJ Andersson B Drews L Duker G Wadstedt G Electrophysiological characterization of the prokinetic agents cisapride and mosapride in vivo and in vitro: Implications for proarrhythmic potential? J Pharmacol Exp Ther 1997 282 1 220 227 9223557

[B40] Vanommeslaeghe K Hatcher E Acharya C Kundu S Zhong S Shim J Darian E Guvench O Lopes P Vorobyov I Mackerell AD Jr CHARMM General Force Field: A Force Field for Drug-Like Molecules Compatible with the CHARMM All-Atom Additive Biological Force Fields J Comput Chem 2010 31 4 671 690 19575467 10.1002/jcc.21367 PMC2888302

[B41] Chen J Seebohm G Sanguinetti MC Position of aromatic residues in the S6 domain, not inactivation, dictates cisapride sensitivity of HERG and eag potassium channels P Natl Acad Sci USA 2002 99 19 12461 12466 10.1073/pnas.192367299 PMC129467 12209010

[B42] Kii Y Ito T Effects of 5-HT4-receptor agonists, cisapride, mosapride citrate, and zacopride, on cardiac action potentials in guinea pig isolated papillary muscles J Cardiovasc Pharm 1997 29 5 670 675 10.1097/00005344-199705000-00016 9213211

[B43] Tetsue Toda YK Ryoichi Kawatsu The 5-HT4 agonists cisapride, mosapride, and CJ-033466, a novel potent compound, exhibit different human ether-a-go-go-related gene (hERG)-blocking activities J Pharmacol Sci 2007 105 207 210 10.1254/jphs.SC0070243 17928736

[B44] Tack J Camilleri M Chang L Chey WD Galligan JJ Lacy BE Muller-Lissner S Quigley EMM Schuurkes J De Maeyer JH Stanghellini V Systematic review: cardiovascular safety profile of 5-HT4 agonists developed for gastrointestinal disorders Aliment Pharm Ther 2012 35 7 745 767 10.1111/j.1365-2036.2012.05011.x PMC3491670 22356640

[B45] Mushiroda T Douya R Takahara E Nagata O The involvement of flavin-containing monooxygenase but not CYP3A4 in metabolism of itopride hydrochloride, a gastroprokinetic agent: Comparison with cisapride and mosapride citrate Drug Metab Dispos 2000 28 10 1231 1237 10997945

[B46] Kirpotina LN Khlebnikov AI Schepetkin IA Ye RD Rabiet MJ Jutila MA Quinn MT Identification of novel small-molecule agonists for human Formyl peptide receptors and pharmacophore models of their recognition Mol Pharmacol 2010 77 2 159 170 10.1124/mol.109.060673 19903830 PMC2812066

