# Supplementary material for: Rehabilitating drug-induced long-QT promoters: In-silico design of hERG-neutral cisapride analogues with retained pharmacological activity
Source: BMC Pharmacol Toxicol. 2014 Mar 8;15:14. doi: 10.1186/2050-6511-15-14 (PMC4016140; doi:10.1186/2050-6511-15-14)
Supplement: Additional file 1 — Summary of Docking, De Novo Design and Modeling studies for cisapride analogues investigated. [file 2050-6511-15-14-S1.doc]

**SUPPLEMENTARY MATERIALS**

Rehabilitating drug-induced long-QT promoters: In-silico design of hERG-neutral cisapride analogues with retained pharmacological activity

Serdar Durdagi^†^,*, Trevor Randal^†^, Henry J Duff^‡^, Sergei Y Noskov^†^,*

**^†^**Institute for Biocomplexity and Informatics, Department of Biological Sciences, University of Calgary, Calgary, Alberta, Canada; **^‡^**Libin Cardiovascular Institute of Alberta, Calgary, Canada.

| Compounds | 2D Structure | FlexX | | AutoDock | | GOLD | | Glide/XP | | |
| --- | --- | --- | --- | --- | --- | --- | --- | --- | --- | --- |
| Cisapride | 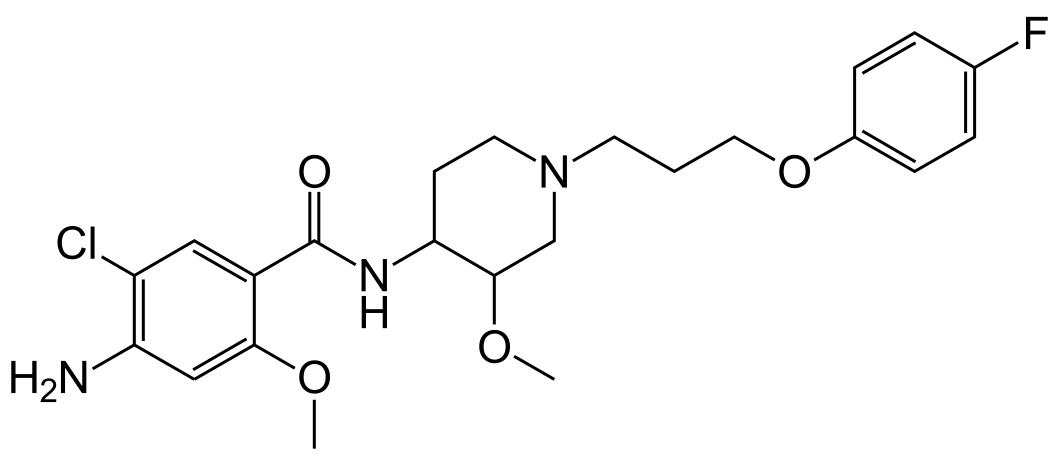 | A2A | hERG | A2A | hERG | A2A | hERG | A2A | hERG | |
|  |  | **-3.97** | **-4.32** | **-6.18** | **-2.72** | **-10.19** | **-9.42** | **-8.48** | **-7.80** | |
| Cisapride-D1 | 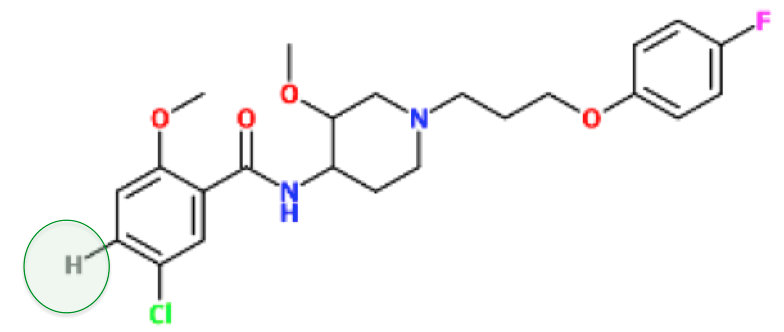 | -4.10 | -4.04 | -5.87 | -2.27 | -10.36 | -8.84 | -9.53 | -7.25 | |
| Cisapride-D2 | 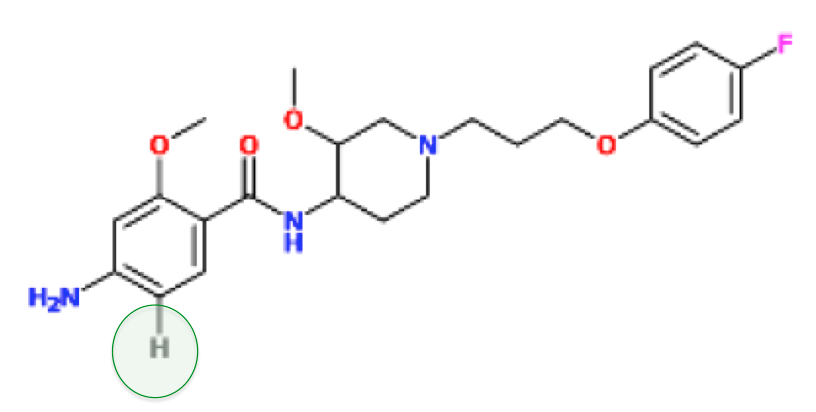 | -4.15 | -4.26 | -5.33 | -1.93 | -9.64 | -8.28 | -5.07 | -7.27 | |
| Cisapride-D3 | 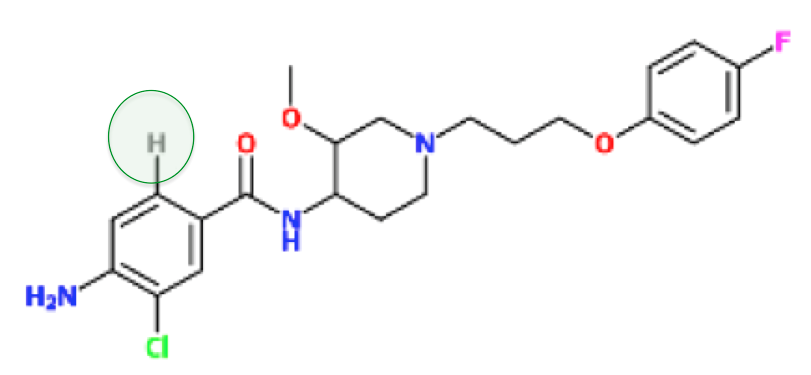 | -4.60 | -5.42 | -4.83 | -1.49 | -9.22 | -8.92 | -8.93 | -7.61 | |
| Cisapride-D4 | 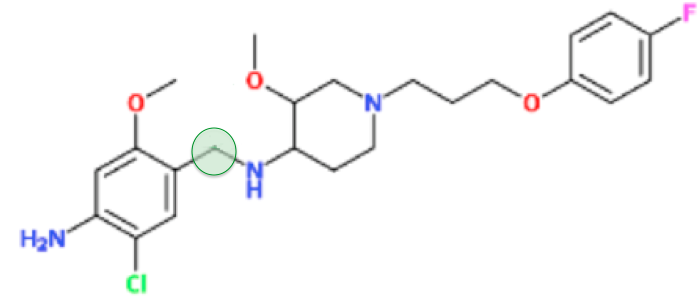 | -4.00 | -3.43 | -5.93 | -1.92 | -10.80 | -9.09 | -7.83 | -7.38 | |
| Cisapride-D5 | 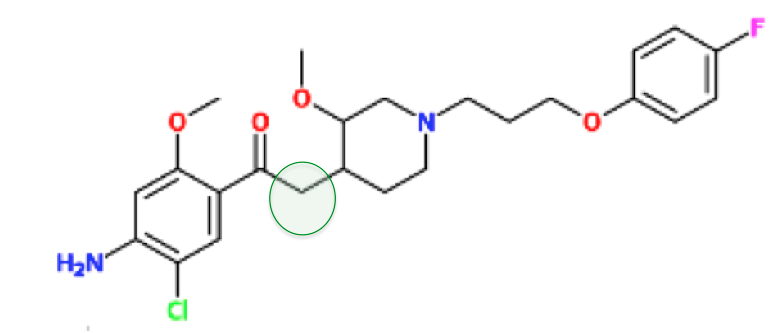 | -4.03 | -3.88 | -5.82 | -2.44 | -10.58 | -8.89 | -9.34 | -7.95 | |
| Cisapride-D6 | 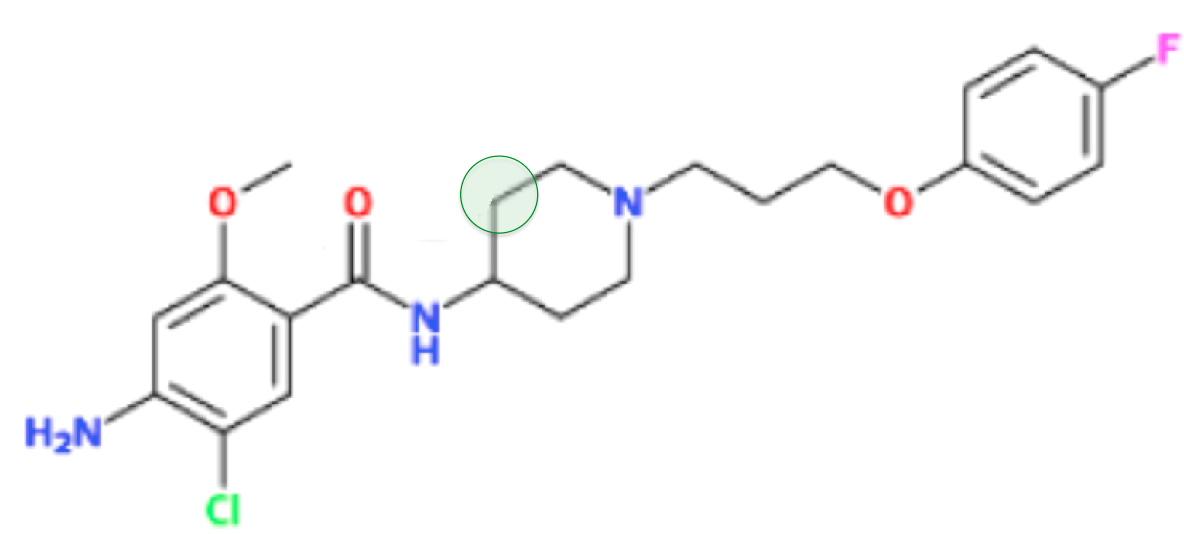 | -3.51 | -4.74 | -7.00 | -2.11 | -9.56 | -8.36 | -7.89 | | -7.01 |
| Cisapride-D7 | 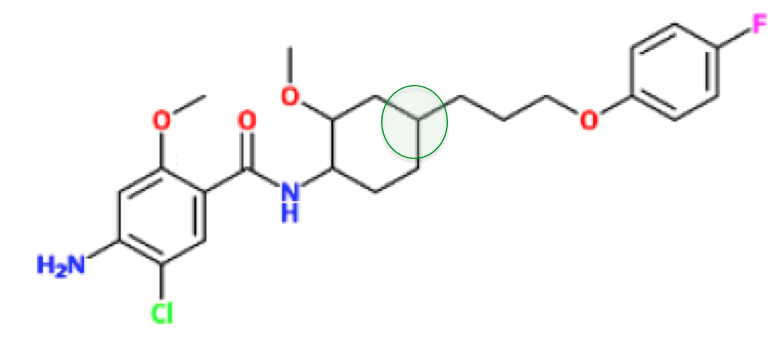 | -4.50 | -4.52 | -6.61 | -2.84 | -10.40 | -8.83 | -8.17 | | -7.46 |
| Cisapride-D8 | 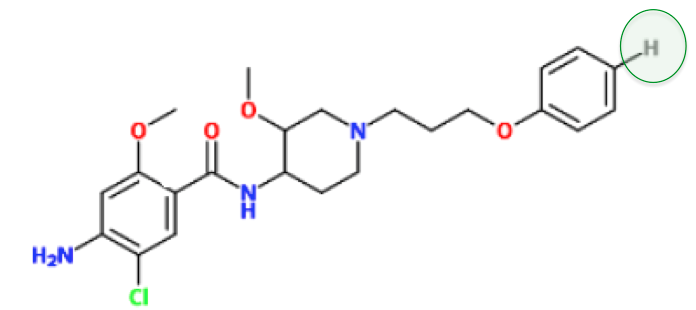 | -4.06 | -4.00 | -7.20 | -2.27 | -10.39 | -9.15 | -9.52 | | -7.10 |
| Cisapride-D9 | 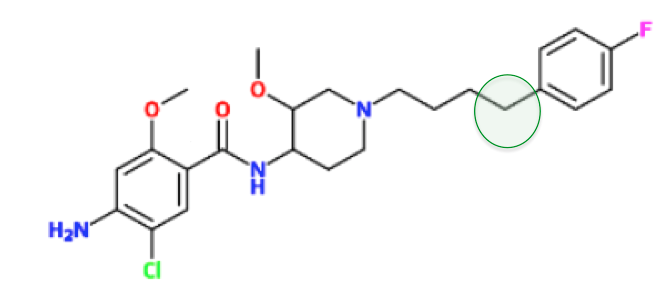 | -4.11 | -4.07 | -5.36 | -1.95 | -10.58 | -9.03 | -9.22 | | -7.45 |
| Cisapride-D10 | 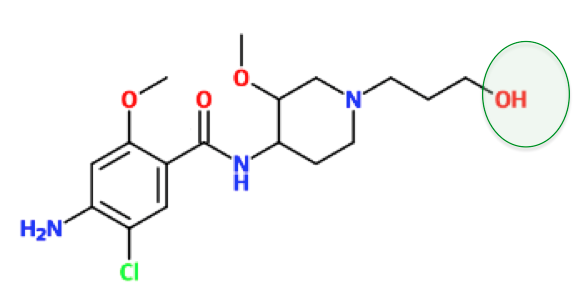 | -3.57 | -3.76 | -3.97 | -0.47 | -10.19 | -7.21 | -8.48 | | NA |
| Cisapride-D11 | 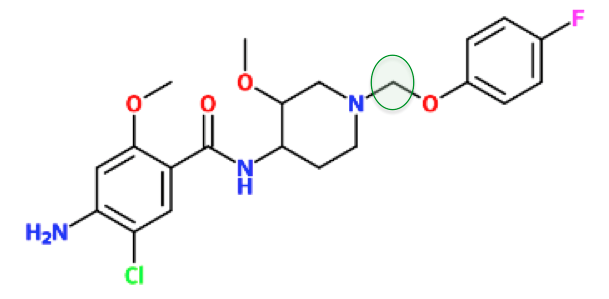 | -3.64 | -4.16 | -5.39 | -2.51 | -9.00 | -7.40 | -9.66 | | -7.09 |
| Cisapride-D12 | 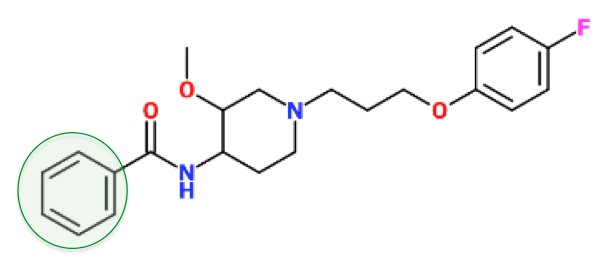 | NA | -4.28 | -4.95 | -1.64 | -10.26 | -8.25 | -7.32 | | -7.00 |

**Table S1.** **Derivation of cisapride analogues.** Several approaches have been considered for the generation of Cisapride derivatives (i.e., change of heteroatoms with hydrogen, decreasing the number of –CH_2_ groups, removing subgroups) to explore the effects of these fragments to the hERG1 blocking and A_2A_ binding scores. (Because of the limited space, only selected derivatives of cisapride have been represented, binding score units are kcal/mol).

| Compounds | 2D Structure | FlexX | | AutoDock | | GOLD | | Glide/XP | |
| --- | --- | --- | --- | --- | --- | --- | --- | --- | --- |
| Cisapride | 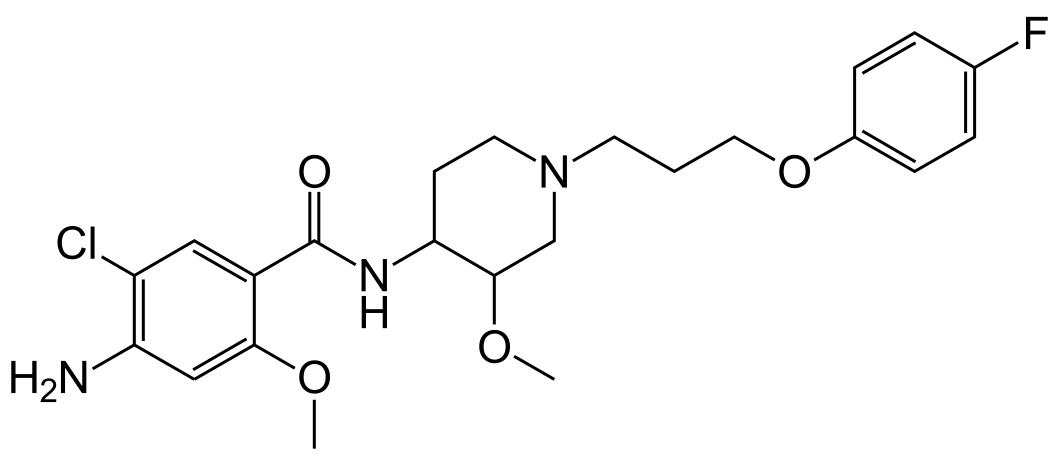 | A2A | hERG | A2A | hERG | A2A | hERG | A2A | hERG |
|  |  | **-3.48** | **-4.32** | **-6.18** | **-2.72** | **-10.19** | **-9.42** | **-8.48** | **-7.80** |
| Ciapride-D11 | 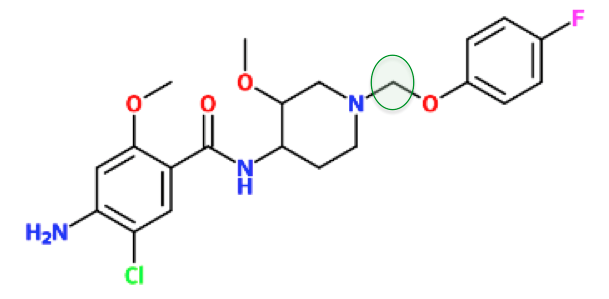 | **-3.64** | **-4.16** | **-5.39** | **-2.51** | **-9.00** | **-7.40** | **-9.66** | **-7.09** |
| Cisapride-D11d1  [Null][Schrodinger_fragment_312_a] | 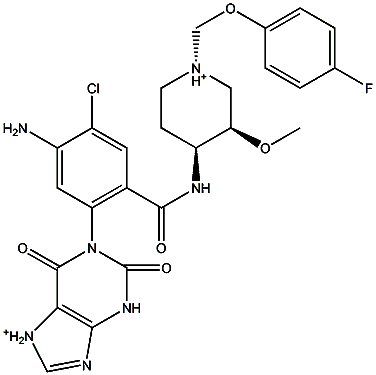 | -4.86 | -4.69 | -1.98 | NA | -9.39 | -7.78 | -9.93 | -5.61 |
| Cisapride-D11d2  [Null][Schrodinger_fragment_317] | 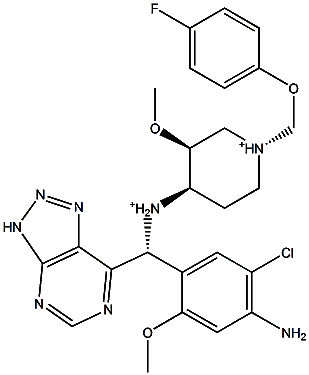 | -4.81 | -3.77 | -1.02 | -1.67 | -9.66 | -7.19 | -8.77 | -5.77 |
| Cisapride-D11d3  [Null][Schrodinger_fragment_111_a] | 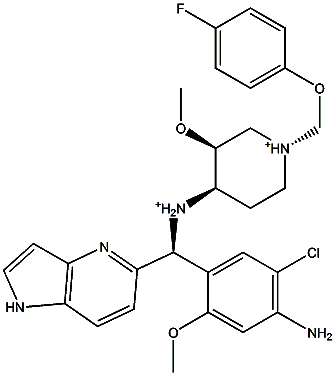 | -5.05 | -4.44 | -4.33 | -2.28 | -12.51 | -9.24 | -8.81 | -5.95 |
| Cisapride-D11d4  [Null][Schrodinger_fragment_391_a] | 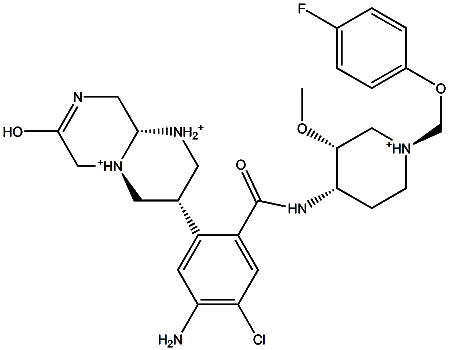 | -3.11 | -4.59 | -3.04 | -0.02 | -9.99 | -8.81 | -8.00 | -6.04 |
| Cisapride-D11d5  [Null][Schrodinger_fragment_306] | 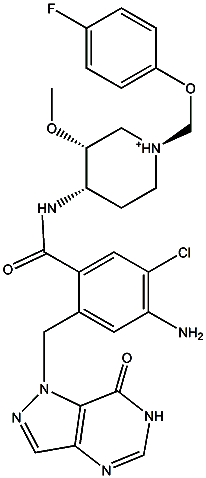 | -4.66 | -4.47 | -4.03 | -1.24 | -10.43 | -8.60 | -9.00 | -6.23 |
| Cisapride-D11d6  [Null][Schrodinger_fragment_206_a] | 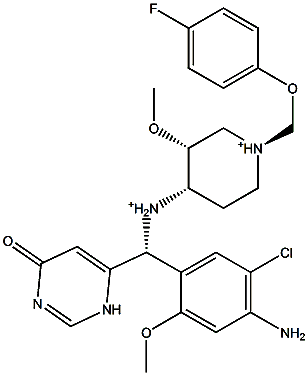 | -2.85 | -3.76 | -1.50 | -2.13 | -10.12 | -7.32 | -8.17 | -6.26 |
| Cisapride-D11d7  [Null][Schrodinger_fragment_261] | 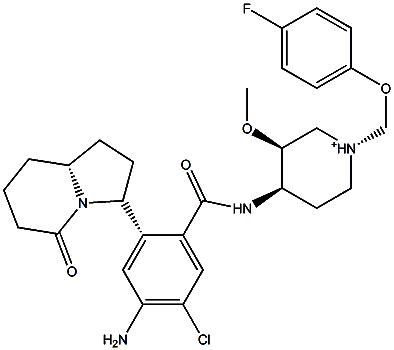 | -4.41 | -3.60 | -6.16 | -3.78 | -11.58 | -8.75 | -8.06 | -6.49 |
| Cisapride-D11d8  [Null][Schrodinger_fragment_406] | 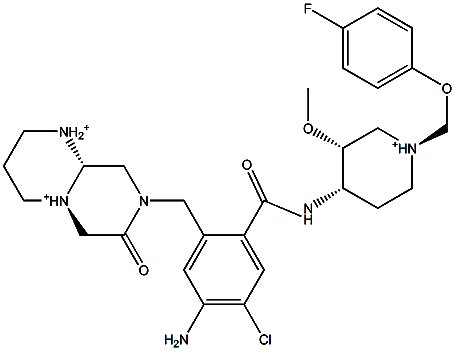 | -3.69 | -5.70 | NA | -1.52 | -9.86 | -8.93 | -9.68 | -6.58 |

**Table S2.** **Derivation of cisapride D-11 analogues.** Cisapride D-11 that includes shorter alkyl chain between terminal aromatic ring and central heterocyclic ring is used as starting structure and it’s analogues are derived using small fragment database of Schrodinger molecular modeling package. Derived compounds are docked at both A_2A_ and hERG receptors. (Because of the limited space, only selected derivatives of cisapride have been represented, binding score units are kcal/mole).


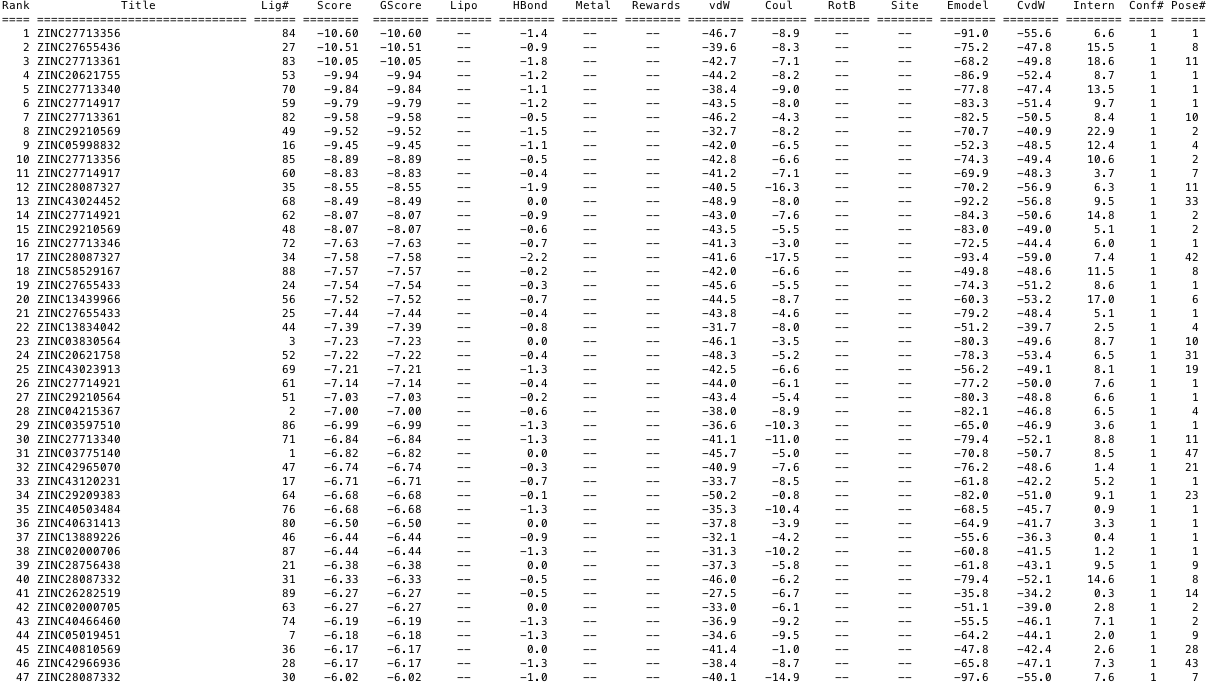


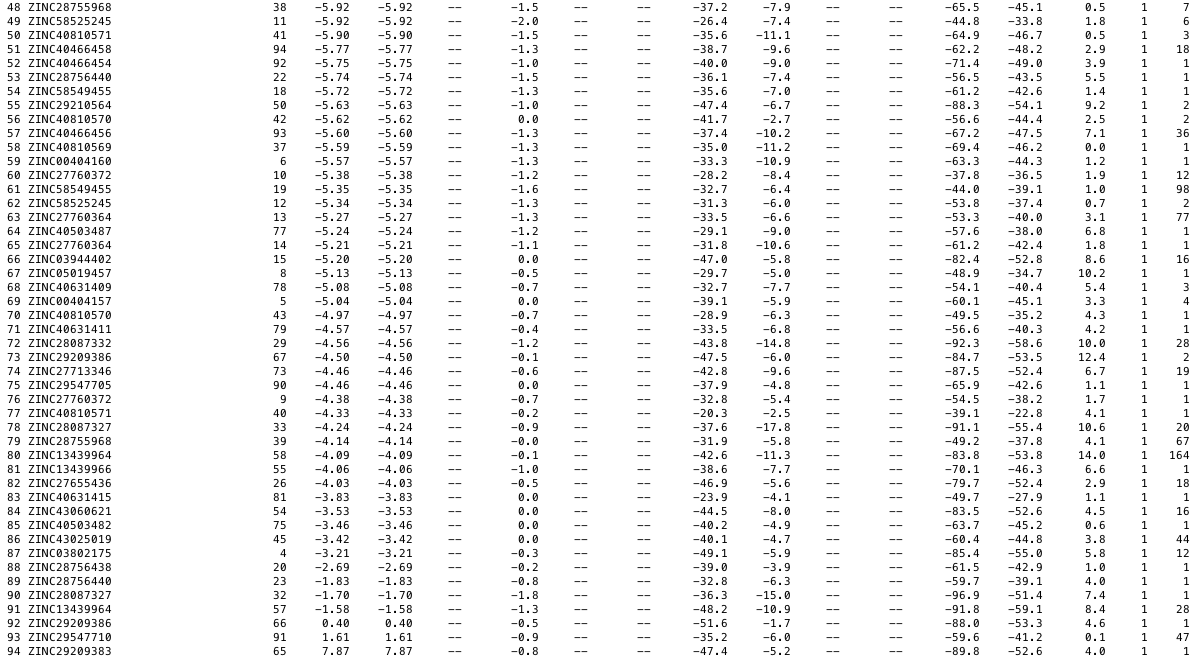


**Table S3.** Docking results of cisapride derivatives from ZINC database at human adenosine A_2A_ receptor.

**
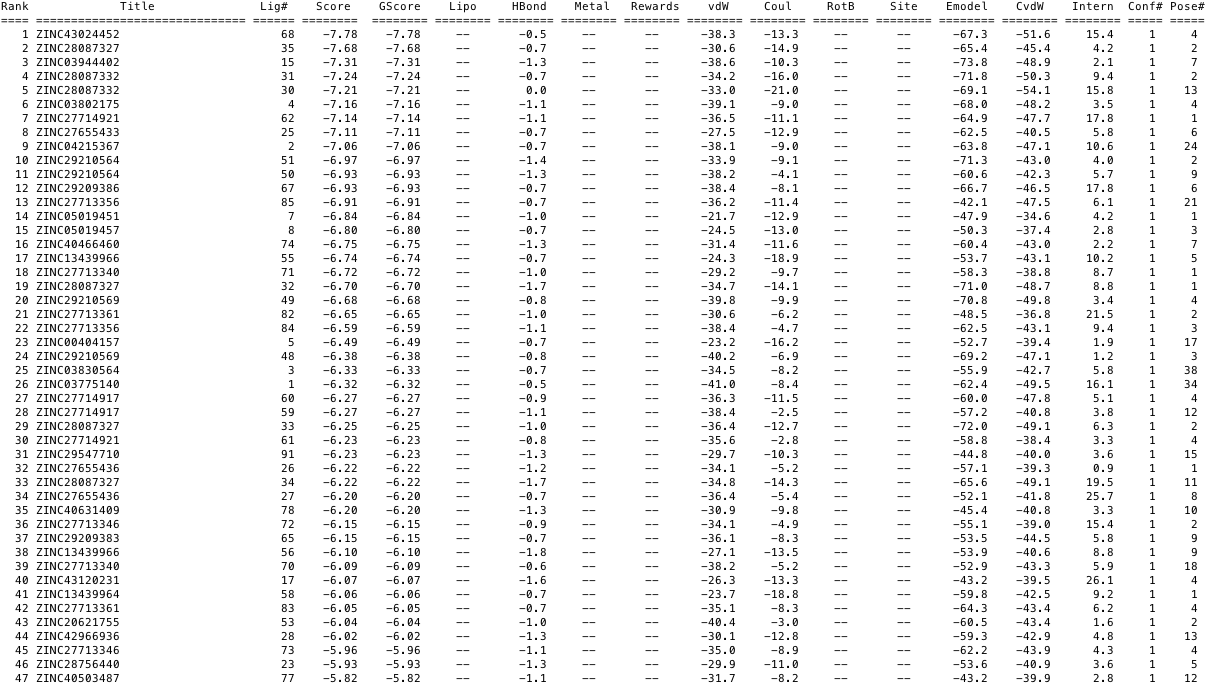
**

**
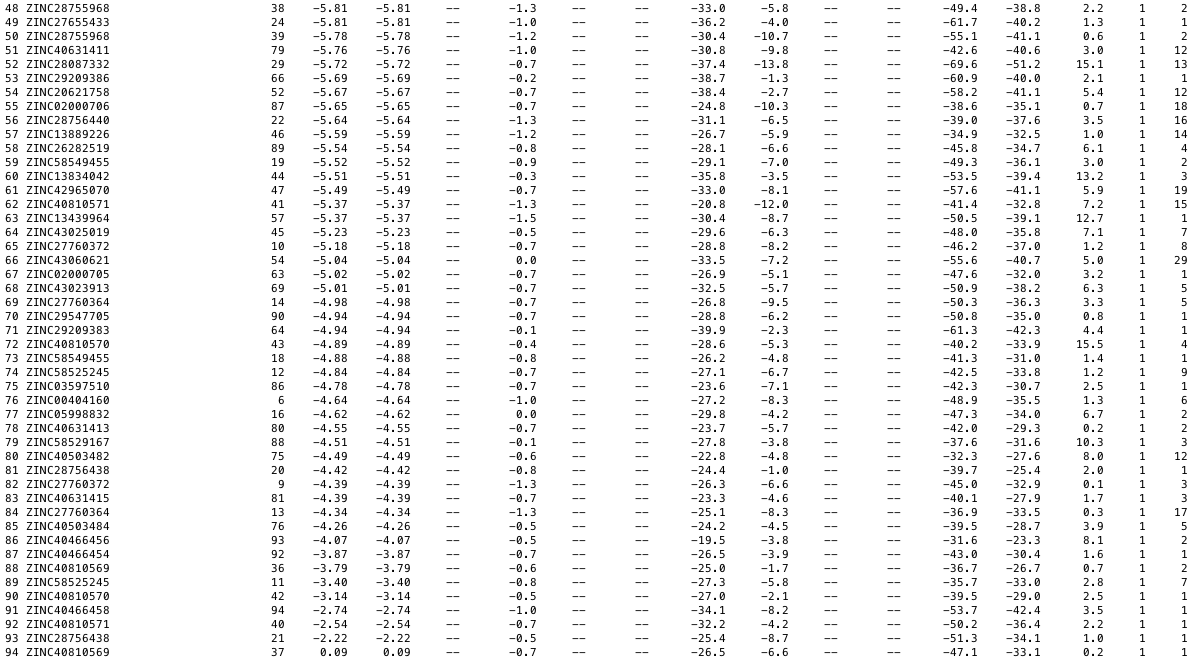
**

**Table S4.** Docking results of cisapride derivatives from ZINC database at hERG1 PD.

| ChEMBL Compounds  Code | 2D Structure | Average  Docking Score  (kcal/mole) |
| --- | --- | --- |
| 1632162 | 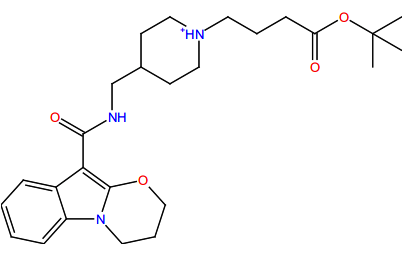 | -6.30 |
| 121913 | 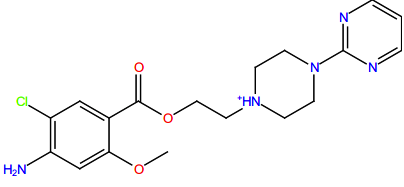 | -6.48 |
| 1632157 | 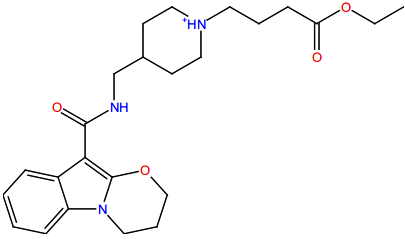 | -6.42 |
| 1632158 | 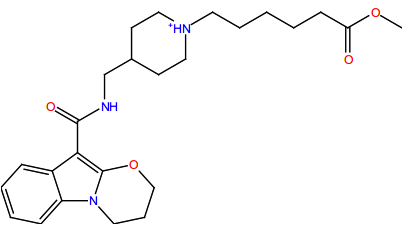 | -6.38 |
| 1632154 | 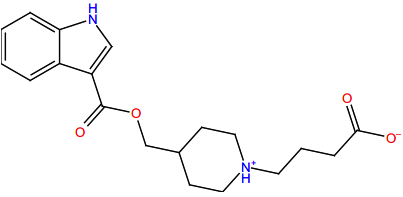 | -6.43 |
| 2179703 | 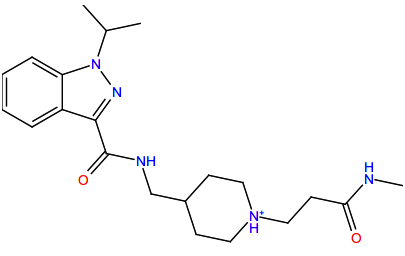 | -6.39 |
| 1632159 | 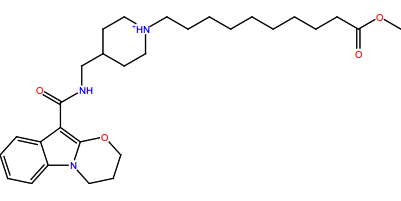 | -6.27 |
| 2179704 | 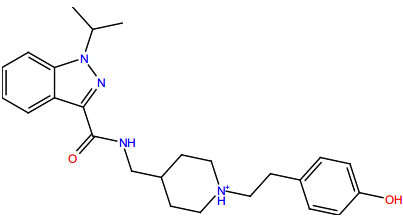 | -6.21 |
| 2181170 | 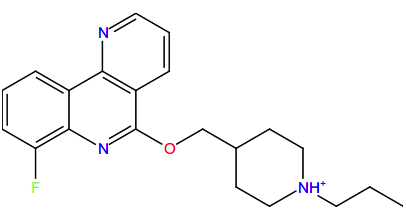 | -5.33 |
| 356359 | 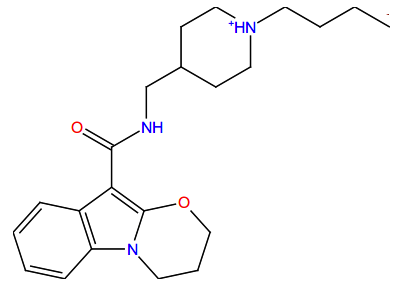 | -6.49 |

**Table S5.** Induced Fit Docking results of compounds at hERG1 PD from ChEMBL database that have better than 1 nM Ki binding affinity at 5HT4 receptor. Results are average values of docking scores for each compounds. (Only compounds that show less than (absolute value) of -6.5 kcal/mole docking score are represented).


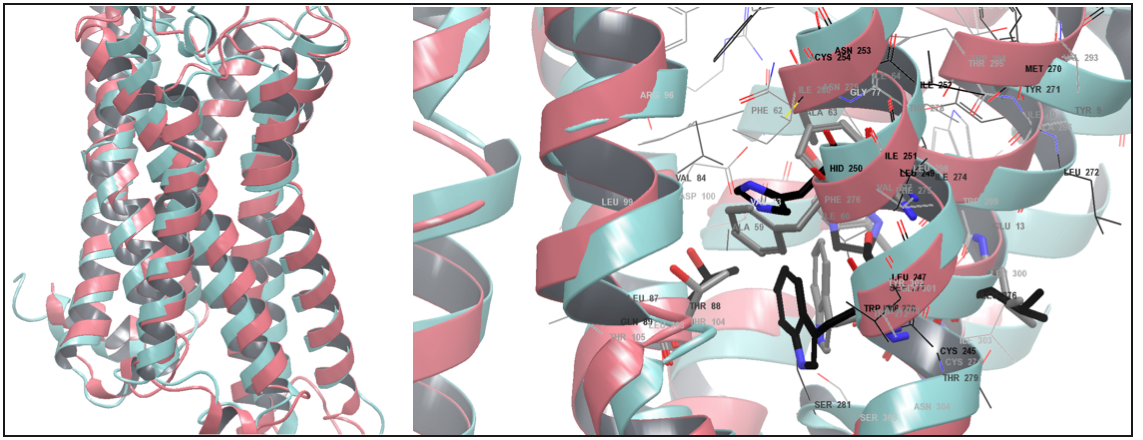


**Figure S1.** (left) Superimposition of X-ray structure of A_2A_ receptor (cyan-colored helices) with 5HT4 modeled receptor (pink-colored helices), (right) binding pocket zoomed.


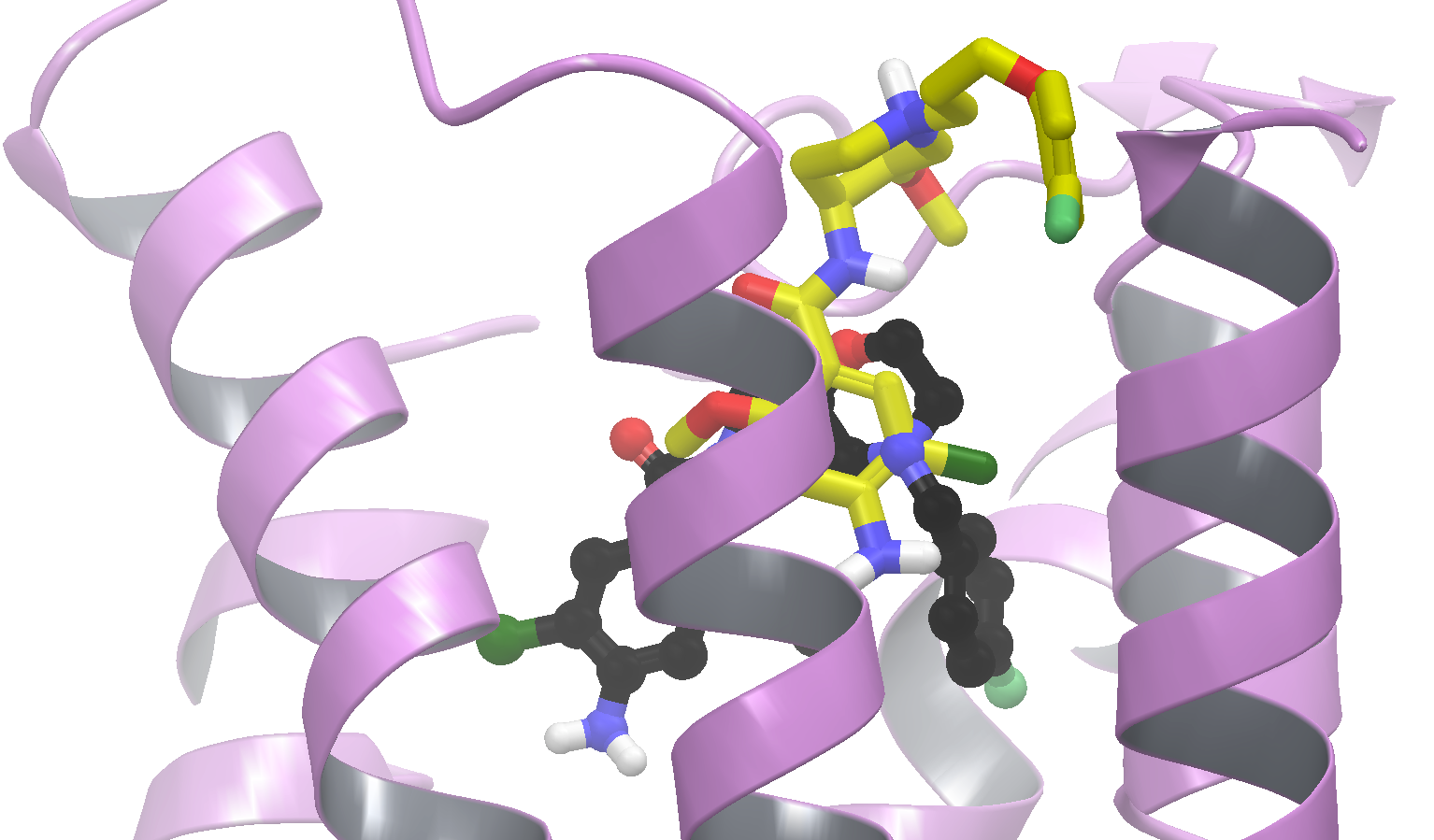


**Figure S2**. Superimposition of top-docking poses of cisapride (shown with yellow-colored carbons) and mosapride (shown with black-colored-carbons).
